# Using whole genome sequence to compare variant callers and breed differences of US sheep

**DOI:** 10.3389/fgene.2022.1060882

**Published:** 2023-01-04

**Authors:** Morgan R. Stegemiller, Reid R. Redden, David R. Notter, Todd Taylor, J. Bret Taylor, Noelle E. Cockett, Michael P. Heaton, Theodore S. Kalbfleisch, Brenda M. Murdoch

**Affiliations:** ^1^ Department of Animal, Veterinary and Food Sciences, University of Idaho, Moscow, ID, United States; ^2^ Texas A&M AgriLife Research and Extension, Texas A&M University, San Angelo, TX, United States; ^3^ School of Animal Sciences, Virginia Tech, Blacksburg, VA, United States; ^4^ Department of Animal and Dairy Sciences, University of Wisconsin-Madison, Madison, WI, United States; ^5^ United States Sheep Experiment Station, United States Department of Agriculture, Agricultural Research Service, Dubois, ID, United States; ^6^ Department of Animal, Dairy and Veterinary Sciences, Utah State University, Logan, UT, United States; ^7^ USDA, ARS, U.S. Meat Animal Research Center, Clay Center, NE, United States; ^8^ Gluck Equine Research Center, College of Agriculture, Food, and Environment, University of Kentucky, Lexington, KY, United States

**Keywords:** sheep, whole genome sequence, freebayes, GATK HaplotypeCaller (HC), variant callers

## Abstract

As whole genome sequence (WGS) data sets have become abundant and widely available, so has the need for variant detection and scoring. The aim of this study was to compare the accuracy of commonly used variant calling programs, Freebayes and GATK HaplotypeCaller (GATK-HC), and to use U.S. sheep WGS data sets to identify novel breed-associated SNPs. Sequence data from 145 sheep consisting of 14 U.S. breeds were filtered and biallelic single nucleotide polymorphisms (SNPs) were retained for genotyping analyses. Genotypes from both programs were compared to each other and to genotypes from bead arrays. The SNPs from WGS were compared to the bead array data with breed heterozygosity, principal component analysis and identifying breed associated SNPs to analyze genetic diversity. The average sequence read depth was 2.78 reads greater with 6.11% more SNPs being identified in Freebayes compared to GATK-HC. The genotype concordance of the variant callers to bead array data was 96.0% and 95.5% for Freebayes and GATK-HC, respectively. Genotyping with WGS identified 10.5 million SNPs from all 145 sheep. This resulted in an 8% increase in measured heterozygosity and greater breed separation in the principal component analysis compared to the bead array analysis. There were 1,849 SNPs identified in only the Romanov sheep where all 10 rams were homozygous for one allele and the remaining 135 sheep from 13 breeds were homozygous for the opposite allele. Both variant calling programs had greater than 95% concordance of SNPs with bead array data, and either was suitably accurate for ovine WGS data sets. The use of WGS SNPs improved the resolution of PCA analysis and was critical for identifying Romanov breed-associated SNPs. Subsets of such SNPs could be used to estimate germplasm composition in animals without pedigree information.

## Introduction

The identification of variants throughout the genome is a critical step in determining which are associated with biological traits. Genotypes from bead arrays are commonly used for analyzing genetic variants that are associated with important traits, however they are typically limited to a selected subset of common variants distributed evenly throughout the genome. Genome sequencing costs continued to decrease significantly over the past 10 years and have enabled the widespread use of whole genome sequence (WGS). Consequently, the amount of genetic information leading to knowledge of genome biology, genetic associations with traits, and improved breeding, has increased (Rexroad et al., 2019).

An advantage of using WGS data for genotyping is the ability to inspect the underlying data for coverage and accuracy as well as identify a large number of SNPs. Genotype accuracy is important for many different analyses including genome-wide association studies, genomic predictions, identification of genetic diseases, and understanding relationships. Berry and others used genotypes from 89 sheep to compare two 50K SNP bead arrays and showed the genotypes were 98% concordant between the two bead arrays, showing that both bead arrays were comparable (Berry et al., 2016). Another study with 31 sheep compared two 50K SNP bead arrays and SNPs identified from WGS analyzed with GATK v4.0 (Marina et al., 2021). They identified similar concordance between the two arrays, 98.8% and slightly lower concordance (95.51%–97.65%) to the genotyping from WGS data (Marina et al., 2021). However, neither study looks at the differences between which variant caller is utilized for analyzing WGS. The current study aims to measure the accuracy of genotypes from WGS in 145 U.S. sheep from 14 breeds by comparing variant discovery and accuracy between two variant callers.

The use of WGS for genotyping samples has increased in prevalence in recent years. A study by Gurgul and others in 2018 used restriction enzymes to genotype by sequence (GBS) samples and demonstrated that increased sequence depth resulted in identifying more SNPs with increased accuracy in livestock species. This study also described how GBS data can accurately be used in analyzing population genetics. Genotypes derived from WGS have been used in other studies to analyze biological traits such as fecundity and brucellosis susceptibility in sheep (Heaton et al., 2017; Nosrati et al., 2019; Li et al., 2021).

Understanding and then maintaining the genetic diversity that exists in domestic livestock breeds is important for the future of agriculture (Rexroad et al., 2019). Recent studies have used WGS to understand and preserve diversity and specific biological traits in breeds that are economically and culturally relevant, including Snow sheep in Siberia and native sheep breeds in Greece (Upadhyay et al. 2021, and Kominakis et al., 2021). Studies that have used bead array genotypes to analyze diversity between global sheep breeds have demonstrated the geographical distribution and differences of breeds from Europe, Asia, and Africa (Kijas et al., 2009 and Kijas et al., 2012). Animals from the same breed but different geographical regions have likewise been shown to be genetically diverse (Kijas et al., 2009 and Davenport et al., 2020). The difference of individuals within the same breed demonstrates that breeding strategies and selection pressures have resulted in genetic variation even within the same breed. Here we use WGS with 15-fold coverage aligned to the ovine reference genome to compare the genotypes from two variant callers Freebayes and GATK HaplotypeCaller (GATK-HC) and analyze genetic diversity from economically important U.S. based sheep breeds. This study found that Freebayes utilized more read depth and identified more SNPs in comparison to GATK-HC. Among the findings provided by these analyses were lists of breed-associated SNPs that could be used to estimate germplasm composition in animals of unknown origin.

## Methods

### Samples

This study used 145 unrelated sheep from 14 U.S. breeds. The breeds were chosen for their relevance to the U.S. sheep industry and consist of different biological phenotypes and utilities such as meat, wool, dairy and hair (Leymaster 1991). The WGS for 95 of the sheep were derived from the 96-member USMARC Sheep Diversity Panel version 2.4 (MSDPv2.4, minus the lone Navajo Churro), the details of which have been published elsewhere (Heaton et al. 2010; Heaton et al. 2017). The remaining animals were collected from private breeders based on their availability and reduced pedigree relationships within breed (Table 1). Blood samples from 50 sheep were collected and DNA from 3 ml of EDTA whole blood was purified with standard procedure that used ammonium chloride lysis, phenol/chloroform extraction, and ethanol precipitation (Sambrook et al., 1989). The purified DNA was dissolved in a solution of 10 mM TrisCl, 1 mM EDTA (TE, pH 8.0), and stored at 4°C. DNA library preparation was conducted as previously described (Heaton et al., 2016). Briefly, DNA was fragmented and indexed for 500 bp paired end libraries. Whole genome shot-gun sequencing was completed with pooled sequencing on the Illumina Next seq 500 with 2 × 150 paired end reads. An average depth of 16.3x coverage was obtained with a minimum of 10x for each sample.

**TABLE 1:** Data from the U.S. sheep breeds used.

Breed	Number of sheep	Sequence coverage^a^	Bead array
MARC III Composite^b^	17	14.36	Illumina
Dorper^b^	10	14.45	Illumina
Dorset^b^	11	16.00	Illumina
East Friesian x Lacaune	10	13.61	Affymetrix
Finn^b^	10	13.61	Illumina
Hampshire	10	14.28	Affymetrix
Katahdin^b^	8	15.08	Illumina
Polypay	10	15.02	Affymetrix
Rambouillet^b^	10	15.79	Illumina
Romanov^b^	10	15.17	Illumina
St. Croix	10	15.78	Affymetrix
Suffolk^b^	9	15.99	Illumina
Targhee	10	16.55	Affymetrix
Texel^b^	10	15.09	Illumina

^a^ Coverage estimated from taking 0.3 x Q20 GB, produced (Heaton et al., 2017).

^b^ Sequence retrieved from U.S., MARC, Cattle and Sheep WGS, site.

The bam files were aligned to the reference assembly Oar_v3.1 using the Burrows-Wheeler Alignment tool (BWA mem 0.7.17). PCR duplicates for these sequences were removed with super deduper v1.0 before alignment (Peterson et al., 2015). Bam files for the 95 sheep were obtained from the sheep reference panel v2.4 on the U.S. MARC Cattle and Sheep WGS site, fastq files were available at NCBI BioProject PRJNA324837 and information about sample mapping and alignment were published previously (Heaton et al., 2017). These samples had PCR duplicates removed with PICARD tools v2.1.1 after alignment (http://broadinstitute.github.io/picard/).

### Variant calling

Two haplotype-based variant detectors were compared in this study: GATK-HC v4.0.2.0 and Freebayes v1.3.1 using default parameters (Van der Auwera and O’Connor, 2020 and Garrison & Marth, 2012). Sequence data were analyzed within each breed cohort to identify genotypes for each animal. Variants identified by Freebayes as multi-nucleotide polymorphisms were transformed to single-nucleotide polymorphisms with vcf-allelic primitives tool in the vcflib v1.0 program (Garrison et al., 2022). The variants from both programs were filtered to retain chromosomal biallelic SNPs with phred quality scores of 20 or greater using bcftools v1.9 (Li 2011). The number of SNPs identified by each caller for each breed were counted using bcftools stats. Mean sequence read depth per genomic location for the SNPs identified for each breed were calculated with vcftools v0.1.16 (Danecek et al., 2011). The mean sequence read depth by breed was visualized in ggplot2 with R v3.6.2.

One consideration for choosing SNPs for bead array panels is that the variant should have a high minor allele frequency, meaning they are present in many breeds and not specific to one breed (Fan et al., 2010). A subset of SNPs identified in all the breeds by Freebayes were combined using vcf-concat in vcftools. For this dataset, even if a SNP was absent in only one breed, the SNP was excluded from the dataset. The 10,521,593 SNPs present in all breeds will further be referred to as the consensus genotypes from WGS data.

### SNP array genotyping

Bead array genotypes were obtained for the sheep to examine the concordance with the genotyping from WGS data. The samples were genotyped on either the Affymetrix 50K array or the Ovine SNP 50 BeadChip (Illumina Inc.) (Table 1). One St. Croix and one Targhee sample could not be genotyped on the bead array, therefore a total of 143 animals were used in the comparisons. Markers that were on both bead array panels were processed as top forward to ensure the consistent strandedness of the variants called from the bead array and the genotypes from WGS data. A total of 40,426 markers from the bead array data were compared to both GATK-HC and Freebayes. Pairwise concordance between the 40,262 genotypes called by the three datasets; bead array, GATK-HC, and Freebayes were calculated using SNP and Variation Suite version 8.7.2 (SVS, Golden Helix, Inc., www.goldenhelix.com.

The percentage of heterozygous SNPs for the bead array data was calculated in SVS and for the consensus genotypes from WGS was calculated using plink v1.9. Briefly, the number of heterozygous SNPs were then divided by the total number of SNPs to obtain the % of heterozygous SNPs per sample.

### Principal component analysis

A principal component analysis (PCA) was performed for both bead array and consensus genotyping from WGS data. This PCA was limited to only U.S. sheep with approximately the same number of unrelated sheep for each of the breeds. Eigenvalues for consensus genotyping from WGS data were calculated in plink v1.9 and for the bead array data in SVS. Then PCA plots were created by plotting the first two eigenvalues for the respective data and both PCA plots were plotted in ggplot2 in R. The plots can then be compared for individual and breed groupings based on the different genetic data.

### Breed associated SNPs in Romanov and St. Croix sheep

Breed associated SNPs as was defined for this study are those having a high frequency of the minor allele (e.g., A2) within the breed, yet an exceedingly low frequency of the same allele among all other breeds. The Romanov and St. Croix breeds were chosen for analyses since their positions in PCA plots suggested they may have breed associated SNP alleles. Bead array data were filtered to retain SNPs with homozygous A2/A2 genotypes in either the Romanov or St. Croix breeds that were heterozygous or homozygous for the opposite allele in all other breeds. Breed associated SNPs from the genotypes from WGS were first filtered to retain SNPs only homozygous in the breed of interest, Romanov or St. Croix. These SNPs were then analyzed in the other breeds with Freebayes variant caller. Only SNPs that were homozygous in the breed of interest, A2, and homozygous for the opposite allele, A1, in all other breeds were counted as breed associated markers. Most SNPs on the bead array chips have a strong ascertainment bias since they were originally chosen based on their high minor allele frequency across breeds. However, the SNPs identified in the WGS data sets used here are not influenced by this bias, and thus may be more useful for identifying breed associated SNPs.

## Results

### Comparison of haplotype-based variant detectors

**FIGURE 1:**
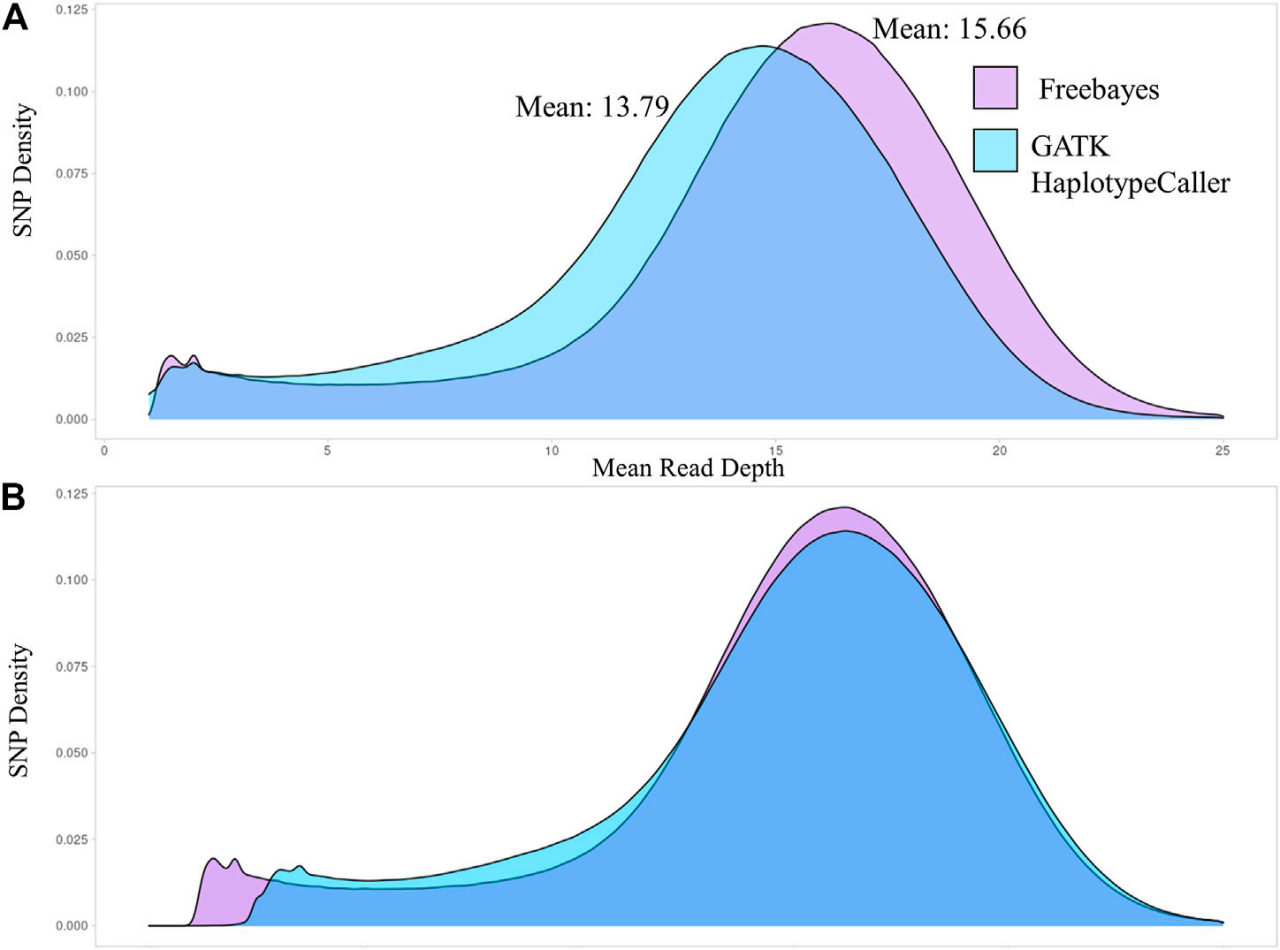
Density distributions of the mean sequence read depth for SNPs identified by Freebayes and GATK-HC for the Hampshire breed. **(A)**. The sequence read depth density curves for both variant callers. **(B)**. The sequence read density curves overlapped with the medians centered.

The number of sequence reads used by each of the variant caller programs to identify SNPs were compared. The distribution curves of the sequence read depth utilized by each variant caller were plotted in Figure 1 using Hampshire as the representative breed and the remaining breeds are shown in Supplemental Figure S1. Freebayes utilized a greater mean sequence read depth than GATK-HC, on average 2.87 greater read depth per breed. A potential reason for this is that when GATK-HC identifies a third allele in a single individual it will not use those reads to identify a variant. The sequence read density distributions had similar shape and were overlapped by adding the difference between the medians to the GATK-HC graph to center the graphs at the medians (Figure 1B). Comparing the number of sequence reads the variant callers used to the number of reads in the bam files confirmed that Freebayes utilized more reads than GATK-HC.

**FIGURE 2:**
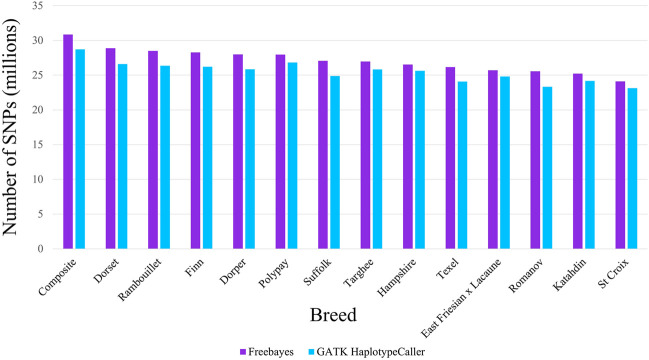
Number of SNPs identified by Freebayes and GATK HaplotypeCaller for each breed cohort.

The SNPs identified by the variant callers were filtered for the analyses which was necessary as Freebayes identified more SNPs with a low-quality score than GATK-HC. After filtering for quality of 20 or greater, freebayes averaged 26.3 million SNPs while GATK-HC still averaged 24.6 million per breed. The number of SNPs identified for the two variant callers were different for all breeds examined. The MARC III Composite had the greatest number of SNPs identified by both variant callers and the St. Croix breed had the least. When listing the breeds from the most to the least number of SNPs, the order was similar between both variant callers. However, the Polypay breed had the largest difference in that it had the sixth most SNPs called by Freebayes but the second most SNP identified by GATK-HC. There were more SNPs detected within breed cohorts by Freebayes than with GATK-HC in all breeds (Figure 2). Freebayes identified an average of 6.11% more SNPs across all breeds, with the largest difference in the Romanov (8.81%) and the smallest in the Hampshire (3.4%) breed. Although Freebayes requires filtering, it identifies more SNPs than GATK-HC in all breeds of sheep.

### Genotype concordance

Pairwise genotype concordance was calculated for both variant callers using WGS and bead array data sets. The concordances of SNPs called by Freebayes and GATK HaplotypeCaller were high and had an average of 99.3% (Table 2). The concordance ranged from 98.94% in the Finn to 99.57% in the Targhee breeds. The variant callers had slightly lower concordance with the bead array genotypic data, Freebayes averaged 96% concordance and GATK-HC 95.5%.

**TABLE 2:** Pairwise concordance of SNP data between both variant callers and bead array.

Breed	Freebayes and bead array^a^	GATK-HC and bead array^a^	Freebayes and GATK-HC
MARC III Composite	96.01	95.53	99.27
Polypay	95.70	94.89	99.23
East Friesian x Lacaune	95.73	95.54	99.52
Targhee	95.87	95.65	99.57
Dorset	96.10	95.60	99.32
Finn	95.91	95.14	98.94
Katahdin	96.02	95.34	99.08
Texel	96.16	95.77	99.46
Hampshire	95.89	95.39	99.56
Suffolk	96.00	95.26	99.03
Rambouillet	96.11	95.61	99.33
Dorper	96.13	95.73	99.41
Romanov	96.10	95.58	99.29
St. Croix	95.92	95.77	99.49
Average	95.98	95.49	99.32

^a^ Concordance was calculated using 40,426 SNPs, from the Illumina and Affymetrix bead arrays called by both variant callers.

**FIGURE 3:**
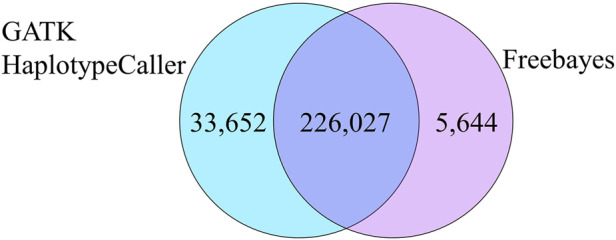
Total non-concordant SNPs that were common and unique between the two variant callers compared to the bead array.

Further comparisons were conducted to analyze the numbers of concordant and non-concordant SNPs from all samples. The largest number of non-concordant SNPs occurred with heterozygous vs. homozygous mismatches (A/B *vs*. A/A or B/B) (Table 3). Homozygous A to homozygous B mismatches (A/A *vs*. B/B) were fewer but were still present in all comparisons (Table 3). There were a total of 226,027 genotypes that neither Freebayes nor GATK-HC matched with the genotype called by the bead array. The Freebayes SNP data had an additional 5,644 genotypes that did not match the bead array genotypes where GATK-HC had concordance. Conversely, there were 33,652 genotypes from GATK-HC data that were non-concordant with the bead array data but matched Freebayes data (Figure 3).

**TABLE 3:** Total number of concordant and non-concordant SNPs between the variant callers and bead array data for all the animals genotyped.

	Freebayes and bead array	GATK-HC^a^ and bead array	Freebayes and GATK-HC^a^
Concordant genotypes	5,542,493	5,508,868	5,814,822
Non-concordant genotypes	231,970	260,018	39,549
Het^b^ vs. Hom^c^ (A/B vs. A/A or B/B)	164,188	188,861	36,431
Hom^c^ A vs. Hom^c^ B (A/A vs. B/B)	67,782	71,157	3,118

^a^ Concordance was calculated using 40,426 SNPs, from both the Illumina and Affymetrix bead arrays and called by both variant callers.

^b^ Heterozygous.

^c^ Homozygous.

Heterozygosity was calculated and analyzed to compare data sets and breeds of sheep. The bead array data had an average heterozygosity of 33.43% (Table 4) for all animals. The overall heterozygosity (41.42%) was greater for consensus genotyping from WGS data. The increase in heterozygosity detected using WGS could be due to having more SNPs (10,521,593) in comparison to the bead array data (40,426). The SNPs present in the consensus genotyping from WGS data were identified in at least one animal in every breed. This shows that there is a deviation from the reference in at least one animal in every breed, so increased heterozygosity in the data set was also expected. Breed heterozygosity rankings of sheep breeds were the same between the two data sets with the exception of the Texel breed. There was a greater difference in the heterozygosity from the consensus WGS genotyping compared to the bead the Texel breed (Table 4).

**TABLE 4:** Comparison of average percent heterozygous SNPs for bead array and consensus genotyping from WGS data.

Breed	Average % heterozygous SNPs		Change in average % heterozygous SNPs
	Bead array	WGS consensus^a^	
Composite	35.87	43.25	7.38
Polypay	35.40	43.06	7.66
East Friesian x Lacaune	35.13	42.62	7.49
Targhee	34.58	42.43	7.85
Dorset	34.40	41.75	7.35
Finn	34.25	41.61	7.36
Katahdin	33.74	41.96	8.22
Hampshire	33.34	40.95	7.61
Texel	33.23	43.31	10.08
Suffolk	32.99	41.05	8.06
Rambouillet	32.50	40.28	7.78
Dorper	32.47	39.78	7.31
Romanov	30.32	39.45	9.13
St. Croix	29.55	38.34	8.79
Average	33.41	41.42	8.01

^a^ The 10.5 Million consensus genotyping from WGS SNPs, identified in all breeds from Freebayes.

### Principal component analysis

**FIGURE 4:**
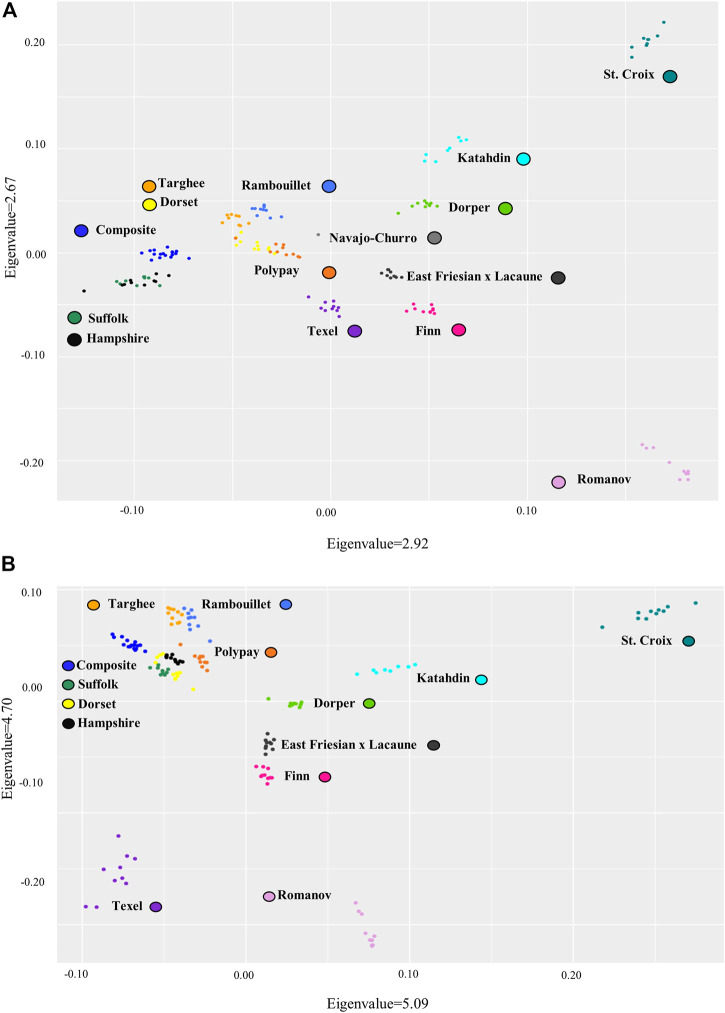
Principal component analysis of variant data for the sheep breeds **(A)**. Bead array variant data and **(B)**. The consensus WGS variant data from Freebayes. Note: MARC III Composite breed is labeled Composite.

Breed relationships were examined by creating PCA plots for both the bead array and consensus genotyping from WGS data sets. Distribution of the breeds in both plots exhibited similarities, although the consensus genotyping from WGS data did have larger eigenvalues (Figure 4). In both plots, the Romanov and St. Croix breeds separate out distinctly. A larger grouping of seven breeds was also present in both plots. The breeds in this grouping consist of MARC III Composite, Targhee, Rambouillet, Polypay, Hampshire, Suffolk, and Dorset. Several of these breeds were used in the formation of some of the others so having them group close together is expected. The Polypay breed was created from crosses among the Targhee, Dorset, Rambouillet, and Finn breeds (Hulet et al., 1984). Rambouillet also contributed to the formation of the Targhee breed (U.S. Targhee Sheep Association). The MARC III Composite was formed using Hampshire, Suffolk, and Columbia (Heaton et al., 2017). Smaller similarities were also retained between the two plots. There is one Polypay that was consistently set farther apart from the rest of the breed clustering. Also, in both plots the Dorset breed has two smaller groupings inside their breed cluster.

Despite the similarities the two PCA plots created from the bead array and consensus WGS data sets had several differences. The Texel separates out from the other breeds farther in the consensus genotyping from WGS data than with the bead array data. The Suffolk and Hampshire grouping is also different between the data sets. In the bead array data set, the Hampshire and Suffolk breeds overlap completely but, in the consensus genotyping from WGS data, the two breeds cluster separately. Overall, the data from WGS and bead array were similar, but the consensus genotyping from WGS data is able to provide more insight into the breed groupings.

### Breed associated SNPs in Romanov and St. Croix sheep

Breed associated SNPs were identified from the bead array data as SNPs where the minor allele (A2) was homozygous in all 10 animals of the associated breed, while all other 135 animals from the remaining 13 breeds were either heterozygous or homozygous for the major allele (A1). From the bead array genotypes there were 1,931 and 1,865 breed associated SNPs identified in the Romanov and St. Croix breeds, respectively. The most stringent filter that could be applied to WGS data retained only SNPs that were homozygous for the minor allele in one breed while all other sheep were homozygous for the opposite allele. In the Romanov breed there were 1,849 SNPs that met these criteria (Supplemental Table S1). Thus, each of these SNPs was homozygous for the minor allele in all 10 Romanov rams (e.g., A2), while all other 135 sheep from the remaining 13 breeds were homozygous for the major allele (e.g., A1). Conversely, there were only 11 of these breed associated SNPs in the St. Croix breed (Supplemental Table S2). These results suggest that breed associated SNPs may be useful in estimating Romanov composition in composite animals and those without pedigree information.

Analysis to identify breed associated SNPs could be influenced by the other breeds included in the analysis. As the St. Croix breed was used to derive the Katahdin breed, keeping the Katahdin breed in the analysis could confound the results as SNPs passed along from the St. Croix to the Katahdin would not show up in the results (Wildeus 1997). Removing the Katahdin breed from the analysis would allow those SNPs to be identified. Removing the Katahdin breed from the St. Croix analysis increased the homozygous SNPs in the St. Croix and not homozygous in any other breed to 2,154 SNPs in the bead array data. Removing the Katahdin with the genotypes from WGS data increased the SNPs associated with the St. Croix to 18.

## Discussion

Whole genome sequencing has and will likely continue to increase in availability. These data should be analyzed to their maximum potential so that variants affecting biological traits can be identified. This study compared the two variant callers, Freebayes and GATK-HC, and examined concordance of the resulting SNP genotypes with bead array data. These data were further utilized to compare the resolution of genotyping from WGS and bead array data in the examination of genetic diversity of U.S. sheep breeds.

Several analyses were conducted to compare the variants called from Freebayes and GATK-HC. The number of reads used by the variant caller to derive a genotype for an animal is part of the vcf record for each SNP and is available by querying the depth attribute of the genotype. In every breed the mean sequence read depth used was greater and identified more SNPs in Freebayes in comparison to GATK-HC. The Haplotype caller performs a local realignment of the reads spanning a polymorphic site, and disqualifies reads if they are low quality, or suggest an artifact such as a third haplotype for the region. The use of fewer reads will likely result in fewer heterozygous calls. GATK-HC utilized fewer sequence reads than Freebayes, which possibly explains why GATK-HC did not identify as many SNPs as Freebayes. Biallelic SNPs called from Freebayes and GATK-HC had similar concordance to those called using the bead arrays and had greater than 99% concordance to each other. As shown in other studies, results from Freebayes needed to be filtered as there were a larger number of low-quality variantss identified using Freebayes than GATK-HC (Hwang et al., 2015). Freebayes identified more SNPs, but also required more processing steps and increased filtering to extract clean genotyping from WGS data.

A comparison of the percentage of heterozygous SNPs for U.S. sheep breeds was performed using SNPs from bead array and consensus genotyping from WGS data. Greater levels of % heterozygous SNPs were observed with the consensus genotyping WGS data which may indicate that the heterozygosity of animals is higher than what has been previously described using bead array data. The level of heterozygosity in Hampshire, Suffolk, and Rambouillet sheep breeds determined using bead array were consistent with those reported in previous studies (Davenport et al., 2020). The % of heterozygous SNPs rankings between the bead array and the consensus genotyping from WGS data stayed relatively consistent for all breeds except the Texel. This agrees with a previous publication that noted that changing the number of SNPs did not significantly change heterozygosity breed rankings (Kijas et al., 2012).

The consensus genotyping from WGS data consisted of about 10.5 million SNPs present across all of the breeds by Freebayes. The St. Croix and Romanov breeds exhibited fewer numbers of SNPs identified by the variant callers and had lower % of heterozygous SNPs in the consensus genotyping from WGS data. The MARC III Composite and Dorset sheep breeds had greater heterozygosity and total SNPs called by these variant callers. However, Rambouillet sheep had a greater number of total SNPs called but less overall % of heterozygous SNPs. Similarly, the East Friesian x Lacaune and Katahdin sheep had fewer total SNPs identified but a greater % of heterozygous SNPs from the consensus genotyping from WGS data.

### Principal component analysis

The breed diversity of these data sets was visualized in the PCA plots from both the bead array and consensus genotyping from WGS data. Although the PCA plots between the two data sets are similar, the consensus genotyping from WGS data improved the resolution of grouping for a few breeds of sheep. The consensus genotyping from WGS data is able to distinguish between the Suffolk and Hampshire breeds whereas the SNPs in the bead array data could not. As these breeds are of similar genetic background, it is hard to distinguish the two breeds. Another observed difference was that the Texel breed was slightly more distinct in the consensus genotyping from WGS data. The reference genome used in this study is OAR_v3.1, a Texel genome. Comparing the animals to a reference genome of a similar breed can ensure correct mapping and reduce reference genome similarity bias.

### Breed associated SNPs in Romanov and St. Croix sheep

The number of SNPs in the consensus genotyping from WGS data was fewer than the number identified in each individual breed. For example, the St. Croix, which had the lowest number of SNPs called (about 24 million), still had about 13.5 million more SNPs called than in consensus genotyping from WGS data (about 10.5 million). The consensus data set identifies many SNPs present in a large variety of breeds; however, this does not exclude that variants only present in certain breeds and not included in the consensus genotyping from WGS data may be associated with biological phenotypes. Identifying variants that are present in one or a few breeds of similar biological types can increase understanding of the potential genetic causes of important physiological traits.

Alleles present in only one breed can be used to identify genetic influences on specific signature traits that breeds have. The Romanov and St. Croix were analyzed for alleles homozygous in their respective breeds (A2) and not present in any other breed. The Romanov breed is originally from Russia and has a distinct background compared to the other breeds used in this study (Deniskova et al., 2018). Identifying and understanding breed specific alleles associated with the Romanov can help to identify genetic influences for traits the breed is known for, such as large litter size.

Another potential reason for the increased number of breed associated SNPs in the Romanov is that the animals in this study could have more completely homozygous SNPs than the St. Croix animals. The animals chosen in this study were unrelated, but only a limited number of animals and breeds were analyzed for breed associated SNPs. Further investigation into the demographics of potentially unique alleles is needed to determine if these alleles are fixed throughout the breed and not present in any other breeds, however these are important regions for future analyses.

## Conclusion

This study compared two different variant calling programs using 15x coverage of WGS of 14 U.S. breeds of sheep. The concordance of genotypes identified by sequencing were compared with bead array data. Freebayes identified more SNPs and utilized more sequence reads than GATK-HC. The concordance of the callers to the bead array data was very similar, although Freebayes’s concordance was slightly higher. The consensus genotyping from WGS data showed increased heterozygosity and better breed cluster separation in the PCA plot. The genotyping from WGS data allowed for greater identification of breed associated SNPs in the Romanov and St. Croix breeds. Although the bead array and genotyping from WGS data have similar PCA plots and heterozygosity rank, the increase in the number of SNPs improved the resolution of the clustering of the closely related breeds. This study demonstrated that both variant callers were comparable and the use of genotyping from WGS improved the number of variants for the identification of genetic diversity.

## Data Availability

The data in this study are deposited on the NCBI SRA Archive with the BioProject accession number PRJNA913135
