# Supplementary material for: Using whole genome sequence to compare variant callers and breed differences of US sheep
Source: Front Genet. 2023 Jan 4;13:1060882. doi: 10.3389/fgene.2022.1060882 (PMC9846548; doi:10.3389/fgene.2022.1060882)
Supplement: Supplementary file 1 [file DataSheet1.docx]

Supplementary Material


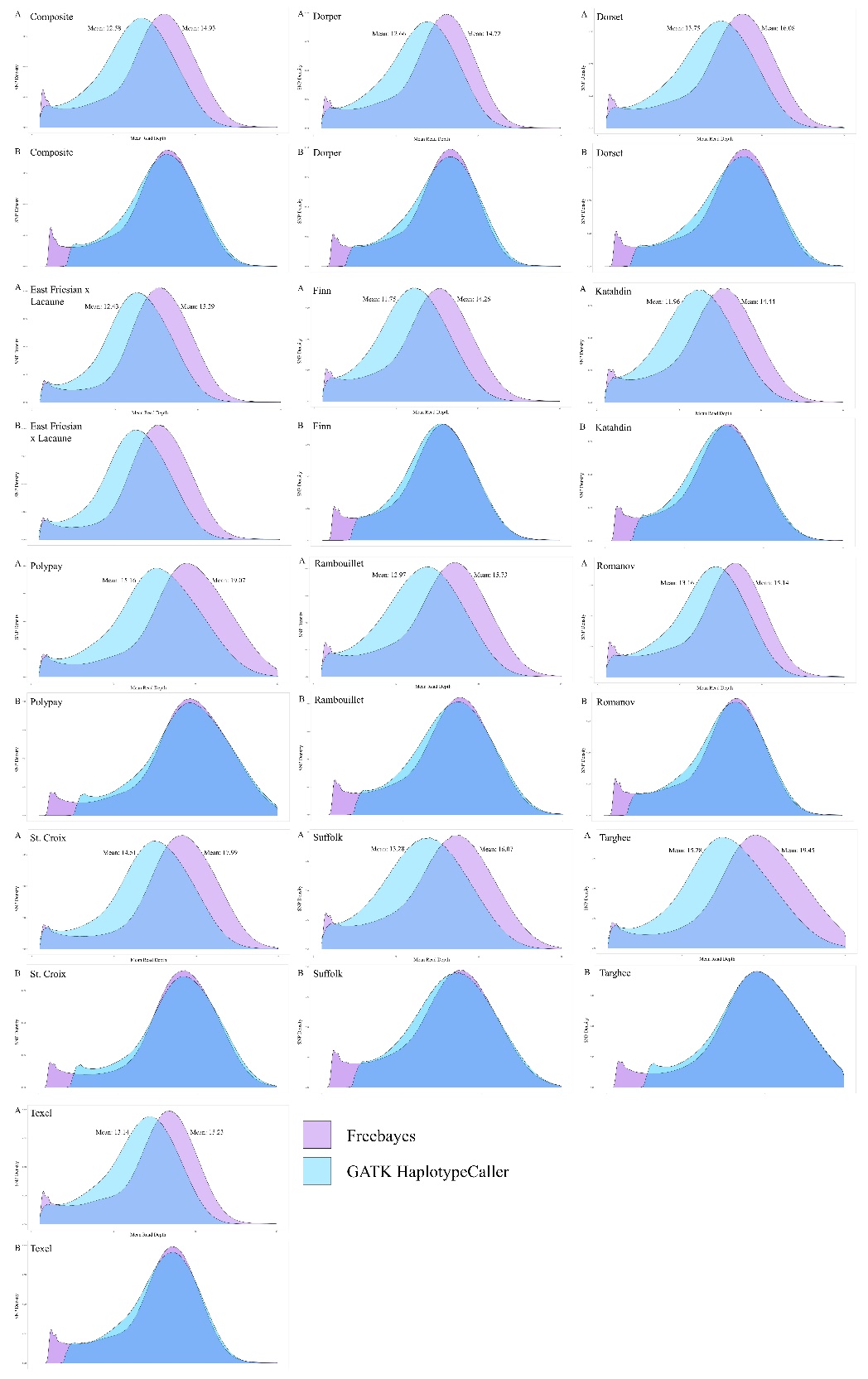


**Supplementary Figure 1. Density distribution of the mean sequence read depth for biallelic SNPs identified by Freebayes and GATK-HC for thirteen breeds. A. The sequence read depth density curves for both variant callers. B. The sequence read density curves overlapped with the medians centered.**

**Supplementary Table 1. Breed associated SNPs identified in the Romanov breed from the whole genome sequence data.**

| Chromosome | Position | Unique allele | Alternate allele |
| --- | --- | --- | --- |
| 1 | 7127670 | A | T |
| 1 | 7259366 | T | G |
| 1 | 10334950 | T | A |
| 1 | 11652844 | G | T |
| 1 | 12066563 | C | A |
| 1 | 14205064 | A | T |
| 1 | 16276491 | T | G |
| 1 | 17193668 | C | A |
| 1 | 17263343 | T | A |
| 1 | 17310836 | A | T |
| 1 | 22490774 | T | C |
| 1 | 23498211 | A | C |
| 1 | 25348755 | C | T |
| 1 | 27985393 | C | T |
| 1 | 30743091 | A | G |
| 1 | 33868707 | C | A |
| 1 | 34442699 | C | T |
| 1 | 38093559 | C | A |
| 1 | 43557206 | A | G |
| 1 | 43573971 | A | C |
| 1 | 43723423 | G | T |
| 1 | 44717803 | G | T |
| 1 | 45409104 | C | G |
| 1 | 45522819 | A | T |
| 1 | 46457690 | A | T |
| 1 | 46971679 | C | T |
| 1 | 47988806 | T | A |
| 1 | 49780622 | A | C |
| 1 | 49929664 | G | T |
| 1 | 54456253 | G | T |
| 1 | 54876956 | G | T |
| 1 | 56234140 | C | G |
| 1 | 57108645 | A | T |
| 1 | 57174431 | G | C |
| 1 | 60016292 | T | A |
| 1 | 60023820 | G | C |
| 1 | 62190915 | C | T |
| 1 | 63626809 | T | A |
| 1 | 67796894 | A | T |
| 1 | 68058363 | A | G |
| 1 | 68633176 | T | A |
| 1 | 69616962 | C | T |
| 1 | 77257205 | G | T |
| 1 | 78304183 | G | T |
| 1 | 78631572 | A | T |
| 1 | 79438393 | T | A |
| 1 | 81396993 | T | C |
| 1 | 81410914 | T | G |
| 1 | 82541076 | G | T |
| 1 | 92432638 | G | C |
| 1 | 92952215 | G | T |
| 1 | 94273549 | G | A |
| 1 | 95381152 | A | T |
| 1 | 95802927 | G | T |
| 1 | 96277250 | C | T |
| 1 | 97084549 | T | A |
| 1 | 97887994 | T | G |
| 1 | 98988362 | A | T |
| 1 | 99555064 | T | A |
| 1 | 100976828 | C | T |
| 1 | 102050522 | A | T |
| 1 | 102491155 | G | C |
| 1 | 102666715 | T | A |
| 1 | 104890263 | T | C |
| 1 | 107171246 | G | C |
| 1 | 108284184 | A | T |
| 1 | 108440565 | T | A |
| 1 | 108700517 | T | A |
| 1 | 110953764 | A | C |
| 1 | 112697855 | G | C |
| 1 | 117033635 | T | A |
| 1 | 118799110 | A | T |
| 1 | 120020882 | T | A |
| 1 | 120706125 | G | C |
| 1 | 122979119 | T | A |
| 1 | 123748442 | A | T |
| 1 | 124102146 | A | T |
| 1 | 126321270 | A | T |
| 1 | 128397816 | A | T |
| 1 | 129268500 | T | G |
| 1 | 130187008 | C | A |
| 1 | 130503716 | T | G |
| 1 | 130931493 | T | A |
| 1 | 131034386 | A | T |
| 1 | 131887176 | C | A |
| 1 | 132728482 | T | A |
| 1 | 133134870 | A | T |
| 1 | 136324313 | A | G |
| 1 | 139050634 | G | A |
| 1 | 139604760 | C | G |
| 1 | 140791996 | A | T |
| 1 | 143336116 | A | G |
| 1 | 144378971 | A | T |
| 1 | 146395482 | T | C |
| 1 | 146552576 | C | A |
| 1 | 148775516 | T | A |
| 1 | 150000482 | G | A |
| 1 | 150125552 | A | T |
| 1 | 150821188 | G | C |
| 1 | 151426339 | A | T |
| 1 | 151532761 | A | C |
| 1 | 151908407 | T | A |
| 1 | 152282004 | A | T |
| 1 | 152441196 | C | A |
| 1 | 152700189 | T | G |
| 1 | 152702947 | C | G |
| 1 | 153507947 | A | T |
| 1 | 153998086 | A | T |
| 1 | 154957911 | G | T |
| 1 | 155761621 | G | C |
| 1 | 157477854 | A | T |
| 1 | 157881476 | G | T |
| 1 | 158687345 | T | G |
| 1 | 161998355 | G | T |
| 1 | 162731523 | C | G |
| 1 | 163535572 | A | T |
| 1 | 164976208 | T | C |
| 1 | 165274624 | C | A |
| 1 | 166307596 | G | C |
| 1 | 166777641 | C | T |
| 1 | 167045514 | T | C |
| 1 | 167253581 | T | A |
| 1 | 168058473 | C | G |
| 1 | 168141798 | C | G |
| 1 | 168906989 | A | G |
| 1 | 169787686 | A | T |
| 1 | 170649075 | G | T |
| 1 | 171235535 | T | C |
| 1 | 172568539 | C | A |
| 1 | 173179319 | G | A |
| 1 | 173513930 | A | C |
| 1 | 175137159 | T | A |
| 1 | 177416877 | A | C |
| 1 | 179116823 | C | G |
| 1 | 179358003 | C | G |
| 1 | 181015417 | A | C |
| 1 | 186743808 | C | T |
| 1 | 187751865 | C | G |
| 1 | 193644051 | T | G |
| 1 | 193979307 | T | A |
| 1 | 194775340 | T | A |
| 1 | 195784344 | C | A |
| 1 | 196208625 | T | A |
| 1 | 198859950 | T | A |
| 1 | 199968415 | T | A |
| 1 | 202760899 | A | T |
| 1 | 205288126 | A | G |
| 1 | 207070738 | G | C |
| 1 | 207222894 | T | A |
| 1 | 209005040 | A | C |
| 1 | 209421817 | A | T |
| 1 | 209846257 | T | A |
| 1 | 210072713 | G | C |
| 1 | 210926771 | T | A |
| 1 | 213653258 | A | T |
| 1 | 215274358 | A | T |
| 1 | 216191770 | C | T |
| 1 | 216462580 | A | T |
| 1 | 218789574 | T | C |
| 1 | 218808565 | A | T |
| 1 | 219126771 | G | A |
| 1 | 219465997 | C | T |
| 1 | 220583915 | A | T |
| 1 | 221631662 | C | A |
| 1 | 221956271 | C | A |
| 1 | 222378272 | T | A |
| 1 | 224316763 | T | A |
| 1 | 225757543 | A | T |
| 1 | 226828203 | G | T |
| 1 | 226877326 | A | T |
| 1 | 228321688 | C | T |
| 1 | 230195903 | T | A |
| 1 | 230976857 | G | T |
| 1 | 233188300 | T | C |
| 1 | 234888084 | C | A |
| 1 | 234977857 | C | T |
| 1 | 235010170 | C | A |
| 1 | 240095870 | A | C |
| 1 | 240907779 | T | G |
| 1 | 241047851 | C | A |
| 1 | 242093736 | T | A |
| 1 | 244540386 | C | G |
| 1 | 244682199 | A | G |
| 1 | 249647442 | A | T |
| 1 | 250561727 | G | C |
| 1 | 252127730 | C | A |
| 1 | 253981651 | T | A |
| 1 | 254359734 | G | C |
| 1 | 254647650 | A | T |
| 1 | 257265419 | T | A |
| 1 | 257558045 | T | C |
| 1 | 265819622 | A | T |
| 1 | 267539514 | C | G |
| 1 | 270581374 | T | A |
| 1 | 273162250 | C | G |
| 2 | 4872566 | C | A |
| 2 | 4959765 | G | T |
| 2 | 5893473 | C | T |
| 2 | 10802328 | T | A |
| 2 | 11338542 | T | A |
| 2 | 12527934 | T | G |
| 2 | 12721438 | A | C |
| 2 | 12803338 | T | C |
| 2 | 13155011 | T | A |
| 2 | 13237805 | A | G |
| 2 | 15105249 | T | A |
| 2 | 16734351 | C | T |
| 2 | 16808284 | C | A |
| 2 | 22778892 | A | G |
| 2 | 22893821 | C | T |
| 2 | 24665431 | T | A |
| 2 | 25110876 | G | T |
| 2 | 25133374 | C | T |
| 2 | 27524211 | G | T |
| 2 | 31086324 | T | A |
| 2 | 32958043 | C | A |
| 2 | 36165450 | A | C |
| 2 | 38470907 | C | G |
| 2 | 41569439 | T | A |
| 2 | 42134459 | A | T |
| 2 | 42140258 | T | A |
| 2 | 46276993 | A | C |
| 2 | 47247757 | T | A |
| 2 | 47528013 | A | T |
| 2 | 47668378 | C | A |
| 2 | 49175520 | C | G |
| 2 | 52475952 | T | G |
| 2 | 55893404 | C | A |
| 2 | 56342648 | G | C |
| 2 | 56972976 | C | A |
| 2 | 57840114 | T | A |
| 2 | 59195004 | G | T |
| 2 | 62283813 | A | T |
| 2 | 62577391 | A | T |
| 2 | 63020425 | T | A |
| 2 | 64400779 | T | A |
| 2 | 64945009 | T | A |
| 2 | 64967882 | C | A |
| 2 | 65788615 | G | T |
| 2 | 65817624 | T | C |
| 2 | 66619005 | A | T |
| 2 | 66883134 | A | G |
| 2 | 67538552 | T | A |
| 2 | 67560310 | A | G |
| 2 | 69157718 | T | C |
| 2 | 69800474 | C | T |
| 2 | 71757096 | A | T |
| 2 | 73808575 | A | G |
| 2 | 77560548 | A | T |
| 2 | 78790896 | A | T |
| 2 | 78888662 | A | G |
| 2 | 79033495 | C | T |
| 2 | 79679531 | C | G |
| 2 | 81272033 | C | G |
| 2 | 83330211 | A | T |
| 2 | 83760822 | T | A |
| 2 | 84823197 | T | A |
| 2 | 86100872 | C | T |
| 2 | 86462551 | T | C |
| 2 | 87749649 | C | A |
| 2 | 88912784 | T | C |
| 2 | 90393768 | T | C |
| 2 | 91290657 | C | A |
| 2 | 91406516 | T | A |
| 2 | 91483335 | T | A |
| 2 | 94263651 | A | G |
| 2 | 96138598 | T | A |
| 2 | 98123878 | A | T |
| 2 | 100130980 | G | T |
| 2 | 101235606 | T | G |
| 2 | 102057301 | T | A |
| 2 | 104314436 | C | T |
| 2 | 105362232 | T | A |
| 2 | 106858674 | A | T |
| 2 | 107798108 | A | C |
| 2 | 112335734 | T | C |
| 2 | 112952778 | C | T |
| 2 | 113031135 | G | T |
| 2 | 113602501 | G | T |
| 2 | 115431960 | A | T |
| 2 | 119284152 | G | T |
| 2 | 121897552 | T | C |
| 2 | 123110477 | T | A |
| 2 | 124739430 | T | A |
| 2 | 125343621 | T | A |
| 2 | 126299633 | A | G |
| 2 | 128623277 | T | A |
| 2 | 129451703 | G | T |
| 2 | 130836885 | T | G |
| 2 | 132219639 | T | G |
| 2 | 134046880 | A | T |
| 2 | 135117827 | T | A |
| 2 | 135734297 | T | G |
| 2 | 137267340 | T | C |
| 2 | 138743373 | T | C |
| 2 | 138769316 | T | C |
| 2 | 138828412 | T | C |
| 2 | 139736096 | T | A |
| 2 | 140000099 | C | T |
| 2 | 142459552 | A | T |
| 2 | 142530911 | T | A |
| 2 | 143166739 | C | G |
| 2 | 143297819 | T | C |
| 2 | 146080050 | T | A |
| 2 | 148853080 | G | C |
| 2 | 150250069 | C | T |
| 2 | 150303164 | C | T |
| 2 | 152312427 | T | A |
| 2 | 153507395 | A | T |
| 2 | 154535884 | C | T |
| 2 | 155400656 | A | C |
| 2 | 157313932 | A | T |
| 2 | 157669432 | T | C |
| 2 | 158279429 | A | C |
| 2 | 160198015 | T | A |
| 2 | 160528795 | T | C |
| 2 | 161018346 | A | T |
| 2 | 161530731 | A | T |
| 2 | 162301672 | G | C |
| 2 | 163856851 | G | T |
| 2 | 165979435 | T | A |
| 2 | 166481881 | C | A |
| 2 | 166543480 | T | C |
| 2 | 168993059 | C | G |
| 2 | 169832089 | C | G |
| 2 | 170222569 | A | T |
| 2 | 170356550 | G | A |
| 2 | 172585168 | C | A |
| 2 | 173133802 | A | T |
| 2 | 173185616 | G | T |
| 2 | 173979050 | T | C |
| 2 | 176430906 | T | A |
| 2 | 178440472 | A | T |
| 2 | 178958658 | G | A |
| 2 | 180766668 | A | C |
| 2 | 181541736 | T | G |
| 2 | 182717547 | T | A |
| 2 | 185774682 | A | T |
| 2 | 186220937 | C | A |
| 2 | 187869815 | C | T |
| 2 | 188031206 | C | G |
| 2 | 188594261 | C | T |
| 2 | 190900687 | A | T |
| 2 | 191360208 | C | T |
| 2 | 193191600 | T | G |
| 2 | 193519351 | A | T |
| 2 | 193647967 | A | G |
| 2 | 194752400 | C | A |
| 2 | 194852975 | T | C |
| 2 | 195883707 | C | T |
| 2 | 196351244 | T | G |
| 2 | 197304891 | T | G |
| 2 | 199629492 | A | T |
| 2 | 199870468 | C | T |
| 2 | 200657360 | T | C |
| 2 | 202360304 | T | A |
| 2 | 204251251 | T | A |
| 2 | 204969987 | T | C |
| 2 | 208011029 | T | A |
| 2 | 210779900 | C | A |
| 2 | 211203469 | C | A |
| 2 | 213867405 | A | T |
| 2 | 213880328 | A | C |
| 2 | 214915003 | C | G |
| 2 | 216306642 | C | T |
| 2 | 216435106 | T | A |
| 2 | 218159194 | A | T |
| 2 | 218174263 | A | C |
| 2 | 218177076 | G | A |
| 2 | 219654198 | A | C |
| 2 | 222140157 | T | A |
| 2 | 223593581 | T | A |
| 2 | 228231841 | T | C |
| 2 | 230620612 | A | C |
| 2 | 230988076 | A | T |
| 2 | 231063787 | G | T |
| 2 | 231123514 | G | T |
| 2 | 231463660 | A | C |
| 2 | 231600484 | C | T |
| 2 | 234336103 | T | C |
| 2 | 236030923 | C | T |
| 2 | 239270683 | T | A |
| 2 | 239372180 | A | T |
| 2 | 244176121 | C | A |
| 3 | 905812 | G | A |
| 3 | 3414002 | C | G |
| 3 | 9208127 | T | G |
| 3 | 9223624 | T | A |
| 3 | 9245069 | G | T |
| 3 | 9871295 | G | T |
| 3 | 12536383 | T | A |
| 3 | 14314728 | C | T |
| 3 | 15180104 | G | T |
| 3 | 15715871 | T | G |
| 3 | 16223002 | G | A |
| 3 | 21529205 | C | G |
| 3 | 21615835 | A | G |
| 3 | 23222787 | C | A |
| 3 | 23623884 | C | G |
| 3 | 23637035 | C | T |
| 3 | 24629064 | A | C |
| 3 | 25178772 | A | T |
| 3 | 25233588 | C | A |
| 3 | 27362405 | A | C |
| 3 | 29662873 | A | C |
| 3 | 29683196 | C | T |
| 3 | 31370805 | T | A |
| 3 | 33360334 | T | C |
| 3 | 33385348 | T | A |
| 3 | 38802189 | A | G |
| 3 | 39098499 | A | T |
| 3 | 39723663 | A | C |
| 3 | 40163690 | A | T |
| 3 | 41346207 | C | T |
| 3 | 42566734 | T | A |
| 3 | 43150468 | C | G |
| 3 | 45275994 | T | A |
| 3 | 45466641 | A | T |
| 3 | 45691993 | G | T |
| 3 | 46207666 | C | T |
| 3 | 46428906 | C | G |
| 3 | 46471941 | G | T |
| 3 | 47267833 | T | C |
| 3 | 47339850 | G | C |
| 3 | 49468637 | T | C |
| 3 | 49892342 | T | G |
| 3 | 51228888 | C | T |
| 3 | 52825536 | T | C |
| 3 | 53224013 | T | A |
| 3 | 53723218 | C | A |
| 3 | 54276382 | T | A |
| 3 | 54551955 | C | A |
| 3 | 54644200 | C | A |
| 3 | 55135568 | G | C |
| 3 | 55222943 | C | T |
| 3 | 55343155 | T | G |
| 3 | 55493141 | T | A |
| 3 | 55871807 | A | T |
| 3 | 61654568 | A | T |
| 3 | 61879528 | G | T |
| 3 | 62212079 | A | T |
| 3 | 62679245 | A | C |
| 3 | 64338462 | G | T |
| 3 | 64699809 | T | A |
| 3 | 66873344 | G | C |
| 3 | 70084876 | T | C |
| 3 | 70257182 | C | A |
| 3 | 72602586 | T | A |
| 3 | 73039584 | A | T |
| 3 | 73974329 | T | G |
| 3 | 74363256 | C | G |
| 3 | 75250607 | T | A |
| 3 | 75677449 | C | G |
| 3 | 76860948 | A | G |
| 3 | 77395578 | T | A |
| 3 | 79683651 | G | C |
| 3 | 80101197 | T | A |
| 3 | 81561769 | C | A |
| 3 | 82544124 | C | G |
| 3 | 83795002 | T | C |
| 3 | 85752217 | T | A |
| 3 | 85918818 | G | T |
| 3 | 86933212 | T | C |
| 3 | 95922351 | T | A |
| 3 | 98637658 | C | T |
| 3 | 99256155 | T | A |
| 3 | 103303571 | G | T |
| 3 | 103766715 | C | T |
| 3 | 104312386 | C | T |
| 3 | 105793376 | C | A |
| 3 | 106489856 | A | G |
| 3 | 109460598 | T | A |
| 3 | 110178115 | C | T |
| 3 | 110692661 | T | A |
| 3 | 112240116 | T | A |
| 3 | 113472020 | C | A |
| 3 | 113859286 | G | T |
| 3 | 117506909 | C | G |
| 3 | 120846182 | C | A |
| 3 | 121758737 | T | C |
| 3 | 122829330 | A | T |
| 3 | 123182523 | A | T |
| 3 | 125850604 | T | C |
| 3 | 127565518 | T | A |
| 3 | 129985375 | A | T |
| 3 | 135657741 | A | T |
| 3 | 137030043 | G | C |
| 3 | 138371615 | T | G |
| 3 | 138688505 | G | A |
| 3 | 139184708 | C | T |
| 3 | 140605864 | G | A |
| 3 | 142798954 | A | G |
| 3 | 144177036 | C | T |
| 3 | 145088685 | G | A |
| 3 | 145772437 | A | G |
| 3 | 146872857 | C | A |
| 3 | 147076401 | T | C |
| 3 | 152942686 | T | G |
| 3 | 158521622 | C | T |
| 3 | 159189923 | A | T |
| 3 | 159605922 | G | T |
| 3 | 163552012 | C | G |
| 3 | 167678763 | T | C |
| 3 | 167905215 | A | T |
| 3 | 170674349 | C | A |
| 3 | 170982757 | G | T |
| 3 | 171547584 | T | A |
| 3 | 175858456 | C | T |
| 3 | 177601011 | A | T |
| 3 | 178979506 | G | C |
| 3 | 181279132 | G | C |
| 3 | 182741767 | A | T |
| 3 | 183247314 | C | G |
| 3 | 183326518 | G | T |
| 3 | 185165704 | T | G |
| 3 | 186140577 | T | G |
| 3 | 186221322 | C | A |
| 3 | 187023495 | A | T |
| 3 | 189572700 | A | C |
| 3 | 190104641 | C | A |
| 3 | 190430949 | G | T |
| 3 | 193250151 | G | T |
| 3 | 193276438 | C | T |
| 3 | 196685991 | T | A |
| 3 | 196727155 | T | A |
| 3 | 199185930 | C | T |
| 3 | 199224696 | C | G |
| 3 | 201527490 | A | G |
| 3 | 201734616 | A | G |
| 3 | 202952947 | T | A |
| 3 | 203678752 | A | T |
| 3 | 203925253 | A | T |
| 3 | 205640165 | A | C |
| 3 | 208970977 | T | G |
| 3 | 209907058 | G | T |
| 3 | 210084302 | G | C |
| 3 | 211790590 | A | T |
| 3 | 224036080 | T | A |
| 4 | 525519 | C | G |
| 4 | 748219 | C | T |
| 4 | 973225 | A | C |
| 4 | 1045605 | G | A |
| 4 | 1078421 | C | G |
| 4 | 1742757 | C | A |
| 4 | 1863586 | G | C |
| 4 | 1933512 | A | T |
| 4 | 1947916 | C | A |
| 4 | 2732532 | A | T |
| 4 | 2885450 | A | T |
| 4 | 2923836 | A | T |
| 4 | 4304465 | T | A |
| 4 | 4369969 | A | C |
| 4 | 6231656 | T | A |
| 4 | 6633245 | T | A |
| 4 | 7640808 | C | G |
| 4 | 10194271 | G | T |
| 4 | 10240596 | T | C |
| 4 | 10293182 | C | A |
| 4 | 10732132 | C | T |
| 4 | 13499222 | T | A |
| 4 | 14138286 | A | G |
| 4 | 17103033 | C | T |
| 4 | 17280509 | A | C |
| 4 | 18413191 | A | T |
| 4 | 19376582 | T | A |
| 4 | 21802214 | A | T |
| 4 | 21978736 | A | C |
| 4 | 22158642 | G | T |
| 4 | 23265820 | G | T |
| 4 | 23368922 | G | T |
| 4 | 23509093 | A | T |
| 4 | 24433747 | A | T |
| 4 | 24760187 | A | T |
| 4 | 25375004 | C | T |
| 4 | 25397252 | G | T |
| 4 | 25419815 | G | C |
| 4 | 28701849 | C | A |
| 4 | 30077136 | C | T |
| 4 | 30845257 | T | G |
| 4 | 31299985 | A | G |
| 4 | 32289859 | T | A |
| 4 | 36040995 | A | T |
| 4 | 36079519 | T | C |
| 4 | 36098434 | A | T |
| 4 | 37640897 | A | T |
| 4 | 38259644 | G | C |
| 4 | 40808369 | G | C |
| 4 | 40883366 | A | T |
| 4 | 41370680 | G | T |
| 4 | 41504253 | T | A |
| 4 | 41676266 | G | A |
| 4 | 43816618 | T | G |
| 4 | 46401317 | T | C |
| 4 | 49778334 | C | A |
| 4 | 50862342 | A | C |
| 4 | 50910942 | T | A |
| 4 | 51123991 | G | A |
| 4 | 54328154 | C | G |
| 4 | 55429022 | A | G |
| 4 | 57449912 | T | A |
| 4 | 58599937 | C | T |
| 4 | 58833325 | C | A |
| 4 | 59002628 | C | G |
| 4 | 59516429 | G | T |
| 4 | 59965622 | A | T |
| 4 | 60220850 | T | A |
| 4 | 60257109 | A | T |
| 4 | 60787978 | A | C |
| 4 | 61793312 | C | T |
| 4 | 61851845 | C | T |
| 4 | 63569095 | A | T |
| 4 | 64442528 | T | A |
| 4 | 70114058 | T | A |
| 4 | 70795650 | T | C |
| 4 | 73588861 | A | T |
| 4 | 73907552 | G | T |
| 4 | 75535304 | T | A |
| 4 | 75717496 | G | T |
| 4 | 80565653 | G | T |
| 4 | 81097972 | T | G |
| 4 | 82454923 | T | A |
| 4 | 83401261 | G | T |
| 4 | 83486561 | A | T |
| 4 | 83632378 | T | A |
| 4 | 84196848 | C | T |
| 4 | 84703369 | G | T |
| 4 | 85876288 | C | G |
| 4 | 88279953 | T | G |
| 4 | 90023160 | A | T |
| 4 | 90086804 | T | C |
| 4 | 90414750 | C | A |
| 4 | 91729918 | A | C |
| 4 | 92616253 | G | T |
| 4 | 94165024 | C | A |
| 4 | 95058519 | C | G |
| 4 | 95779106 | T | A |
| 4 | 97512067 | G | T |
| 4 | 97829281 | G | T |
| 4 | 97964011 | A | C |
| 4 | 98345110 | C | A |
| 4 | 100522714 | A | C |
| 4 | 100820792 | T | A |
| 4 | 101131590 | T | C |
| 4 | 102579620 | A | T |
| 4 | 104641147 | T | C |
| 4 | 106392422 | A | T |
| 4 | 108530208 | T | A |
| 4 | 109153365 | C | A |
| 4 | 109261740 | G | A |
| 4 | 109811773 | G | A |
| 4 | 109836488 | C | G |
| 4 | 110625322 | C | T |
| 4 | 112012187 | C | A |
| 4 | 112443762 | C | A |
| 4 | 115856225 | T | A |
| 4 | 116408870 | T | C |
| 5 | 9596148 | T | A |
| 5 | 9727365 | C | T |
| 5 | 11121365 | A | G |
| 5 | 17211096 | G | A |
| 5 | 24467246 | T | A |
| 5 | 25017174 | A | T |
| 5 | 26388163 | G | T |
| 5 | 26410107 | T | G |
| 5 | 26679846 | T | A |
| 5 | 28603085 | T | A |
| 5 | 29750894 | A | T |
| 5 | 32099713 | A | T |
| 5 | 33540399 | A | T |
| 5 | 34130260 | T | C |
| 5 | 35371781 | G | C |
| 5 | 37899697 | C | A |
| 5 | 40278777 | A | T |
| 5 | 44956545 | C | G |
| 5 | 48679546 | C | A |
| 5 | 50545552 | C | T |
| 5 | 53050355 | A | T |
| 5 | 53286838 | A | C |
| 5 | 53790635 | T | A |
| 5 | 54072453 | C | A |
| 5 | 70393340 | G | T |
| 5 | 71649899 | T | G |
| 5 | 72246863 | G | T |
| 5 | 73280030 | T | A |
| 5 | 74567354 | G | T |
| 5 | 74715285 | A | T |
| 5 | 76762549 | A | G |
| 5 | 77693256 | G | A |
| 5 | 82071011 | C | A |
| 5 | 82912659 | T | G |
| 5 | 82986587 | G | A |
| 5 | 84575634 | A | T |
| 5 | 84587964 | C | A |
| 5 | 85965795 | T | G |
| 5 | 86416383 | C | T |
| 5 | 87764828 | A | T |
| 5 | 88101111 | T | A |
| 5 | 88892599 | A | G |
| 5 | 88965641 | A | T |
| 5 | 90310427 | T | G |
| 5 | 91206774 | A | C |
| 5 | 92763514 | C | A |
| 5 | 93119586 | T | C |
| 5 | 95381949 | T | C |
| 5 | 95569336 | A | T |
| 5 | 95624398 | A | T |
| 5 | 96511545 | G | A |
| 5 | 96769011 | A | T |
| 5 | 97009094 | A | G |
| 5 | 97022096 | C | A |
| 5 | 97706197 | C | A |
| 5 | 98409212 | A | T |
| 5 | 100491640 | T | A |
| 5 | 100517274 | A | T |
| 5 | 100806512 | T | A |
| 5 | 102509863 | G | T |
| 6 | 1793304 | T | A |
| 6 | 3158272 | T | G |
| 6 | 8411132 | A | C |
| 6 | 8424855 | G | T |
| 6 | 8988145 | C | G |
| 6 | 9098382 | G | T |
| 6 | 9108821 | T | C |
| 6 | 10904768 | G | C |
| 6 | 12408260 | G | T |
| 6 | 13176447 | C | T |
| 6 | 13427557 | C | T |
| 6 | 13864159 | T | C |
| 6 | 16582656 | A | T |
| 6 | 16604886 | T | C |
| 6 | 17478018 | C | A |
| 6 | 18646291 | C | T |
| 6 | 21404286 | T | A |
| 6 | 21471017 | T | A |
| 6 | 21908505 | T | A |
| 6 | 22277791 | T | C |
| 6 | 22499873 | T | C |
| 6 | 27083877 | T | A |
| 6 | 27750519 | T | G |
| 6 | 28023921 | A | T |
| 6 | 28031247 | A | C |
| 6 | 31173170 | C | A |
| 6 | 33559893 | A | T |
| 6 | 36622849 | T | A |
| 6 | 38805744 | C | T |
| 6 | 40843056 | C | T |
| 6 | 41197434 | T | A |
| 6 | 43092506 | G | T |
| 6 | 43331607 | A | C |
| 6 | 45500431 | C | A |
| 6 | 45919066 | A | T |
| 6 | 46025671 | A | T |
| 6 | 46113485 | T | G |
| 6 | 47324213 | G | T |
| 6 | 48123398 | T | A |
| 6 | 48770632 | A | T |
| 6 | 48974243 | A | T |
| 6 | 49081985 | C | G |
| 6 | 50249893 | A | T |
| 6 | 51598015 | A | C |
| 6 | 51614194 | A | T |
| 6 | 52325764 | C | A |
| 6 | 52374053 | T | A |
| 6 | 53902038 | A | C |
| 6 | 54067883 | T | C |
| 6 | 54292071 | A | C |
| 6 | 56235816 | C | A |
| 6 | 60546680 | C | A |
| 6 | 61510516 | G | C |
| 6 | 62189745 | G | T |
| 6 | 63057636 | A | T |
| 6 | 69498371 | A | C |
| 6 | 70263387 | T | A |
| 6 | 73547720 | C | A |
| 6 | 75219644 | A | T |
| 6 | 75535994 | T | C |
| 6 | 75615659 | T | C |
| 6 | 76015079 | C | A |
| 6 | 76541697 | C | G |
| 6 | 77581120 | C | T |
| 6 | 78336457 | C | T |
| 6 | 78519679 | G | T |
| 6 | 79034248 | G | T |
| 6 | 79825758 | T | G |
| 6 | 81512100 | G | T |
| 6 | 81528465 | A | C |
| 6 | 82140422 | T | A |
| 6 | 82788857 | T | A |
| 6 | 83049259 | A | C |
| 6 | 84504633 | A | T |
| 6 | 85503175 | T | G |
| 6 | 86024563 | T | A |
| 6 | 86340904 | C | G |
| 6 | 86603911 | T | C |
| 6 | 88834826 | A | C |
| 6 | 89392632 | T | G |
| 6 | 91781577 | T | C |
| 6 | 93014966 | T | A |
| 6 | 94380094 | T | A |
| 6 | 94647968 | T | C |
| 6 | 96248907 | G | C |
| 6 | 98821658 | A | G |
| 6 | 101713327 | C | T |
| 6 | 102703362 | C | T |
| 6 | 104686277 | T | C |
| 6 | 106055084 | A | T |
| 6 | 108434061 | G | T |
| 6 | 109746351 | C | G |
| 6 | 110330843 | T | C |
| 6 | 110657152 | A | T |
| 6 | 111026223 | T | G |
| 6 | 111535760 | C | G |
| 6 | 111788380 | C | A |
| 6 | 112260045 | A | T |
| 6 | 112671485 | T | A |
| 6 | 115038447 | T | C |
| 7 | 277060 | A | T |
| 7 | 1259925 | A | C |
| 7 | 3194649 | C | T |
| 7 | 7077981 | T | A |
| 7 | 8233282 | T | A |
| 7 | 10880238 | A | C |
| 7 | 13675381 | T | A |
| 7 | 14308592 | A | C |
| 7 | 16569763 | A | T |
| 7 | 18547664 | A | C |
| 7 | 18639910 | T | A |
| 7 | 22746760 | A | C |
| 7 | 23082259 | C | T |
| 7 | 24414627 | G | T |
| 7 | 24943283 | T | A |
| 7 | 26681812 | T | C |
| 7 | 26954800 | T | C |
| 7 | 27572665 | A | G |
| 7 | 30251657 | A | T |
| 7 | 30477755 | T | A |
| 7 | 30728224 | A | T |
| 7 | 31188497 | T | A |
| 7 | 31761774 | C | A |
| 7 | 31770460 | T | G |
| 7 | 33137712 | C | T |
| 7 | 33912677 | C | A |
| 7 | 36836408 | T | A |
| 7 | 39267590 | T | A |
| 7 | 39587608 | C | A |
| 7 | 39903032 | A | T |
| 7 | 43468186 | A | C |
| 7 | 45041733 | G | T |
| 7 | 45419619 | C | T |
| 7 | 45449427 | A | C |
| 7 | 50299594 | C | A |
| 7 | 50367273 | T | A |
| 7 | 51768560 | A | T |
| 7 | 55252577 | G | T |
| 7 | 55457516 | A | C |
| 7 | 55721772 | G | T |
| 7 | 58089124 | C | T |
| 7 | 58933143 | T | A |
| 7 | 59038591 | C | T |
| 7 | 59967247 | G | C |
| 7 | 59990797 | A | C |
| 7 | 60225771 | A | T |
| 7 | 60360007 | A | G |
| 7 | 61463079 | A | T |
| 7 | 62493288 | A | G |
| 7 | 62523895 | T | A |
| 7 | 62734790 | C | A |
| 7 | 63799890 | A | G |
| 7 | 64951473 | A | C |
| 7 | 66657787 | T | C |
| 7 | 70765442 | T | A |
| 7 | 71266365 | C | T |
| 7 | 73284997 | A | T |
| 7 | 73754539 | T | A |
| 7 | 75830281 | C | A |
| 7 | 82121417 | C | G |
| 7 | 82882539 | C | A |
| 7 | 82930185 | T | C |
| 7 | 90237438 | A | C |
| 7 | 90306099 | T | A |
| 7 | 90345517 | T | A |
| 7 | 90388810 | C | G |
| 7 | 90416244 | T | A |
| 7 | 91353306 | T | A |
| 7 | 91620826 | A | T |
| 7 | 91714549 | A | T |
| 7 | 91772102 | A | T |
| 7 | 93805886 | T | A |
| 7 | 94025086 | C | A |
| 7 | 94774550 | C | T |
| 7 | 95949610 | A | T |
| 7 | 98666585 | C | T |
| 8 | 3507989 | A | T |
| 8 | 5296293 | A | C |
| 8 | 6039664 | G | T |
| 8 | 7209115 | A | C |
| 8 | 7824457 | T | C |
| 8 | 8194157 | A | C |
| 8 | 8504701 | T | C |
| 8 | 8655639 | T | C |
| 8 | 8679967 | A | T |
| 8 | 8699971 | C | A |
| 8 | 8715350 | T | A |
| 8 | 9111837 | G | C |
| 8 | 10558384 | T | A |
| 8 | 12060853 | A | C |
| 8 | 12522034 | G | C |
| 8 | 13813839 | T | C |
| 8 | 16197293 | G | C |
| 8 | 17786186 | G | A |
| 8 | 19087699 | A | C |
| 8 | 19376637 | C | G |
| 8 | 20565214 | T | G |
| 8 | 21269391 | A | T |
| 8 | 22106957 | C | T |
| 8 | 23967465 | G | T |
| 8 | 24490236 | C | A |
| 8 | 24498553 | T | A |
| 8 | 25527340 | C | G |
| 8 | 25645301 | T | A |
| 8 | 27212179 | A | C |
| 8 | 27836354 | T | A |
| 8 | 31081625 | G | T |
| 8 | 31875760 | C | T |
| 8 | 33631077 | T | C |
| 8 | 33983257 | T | A |
| 8 | 34003238 | T | A |
| 8 | 34144074 | C | T |
| 8 | 34454598 | A | G |
| 8 | 34939983 | T | C |
| 8 | 35076807 | A | T |
| 8 | 35822255 | A | T |
| 8 | 38219247 | C | A |
| 8 | 39528367 | T | G |
| 8 | 41243331 | A | T |
| 8 | 41252560 | T | A |
| 8 | 42474529 | A | C |
| 8 | 42550483 | A | T |
| 8 | 42741068 | A | C |
| 8 | 42895775 | A | T |
| 8 | 43026637 | T | A |
| 8 | 43153391 | T | C |
| 8 | 43300357 | G | C |
| 8 | 44749965 | C | T |
| 8 | 46816375 | A | T |
| 8 | 47478275 | T | G |
| 8 | 47566479 | A | T |
| 8 | 48171908 | T | G |
| 8 | 48472062 | A | T |
| 8 | 48636464 | T | A |
| 8 | 50207711 | T | A |
| 8 | 50453669 | C | A |
| 8 | 53147962 | A | C |
| 8 | 53627455 | T | A |
| 8 | 55894448 | T | A |
| 8 | 57625210 | G | C |
| 8 | 59822594 | T | A |
| 8 | 61851802 | C | G |
| 8 | 63712570 | A | C |
| 8 | 64418094 | A | C |
| 8 | 67280147 | T | A |
| 8 | 67780026 | C | G |
| 8 | 68380703 | A | C |
| 8 | 68954677 | A | T |
| 8 | 68984327 | G | T |
| 8 | 70198604 | C | G |
| 8 | 74863775 | C | A |
| 8 | 79040656 | T | A |
| 8 | 84159073 | A | C |
| 8 | 84575346 | A | C |
| 8 | 85746380 | A | T |
| 8 | 86515504 | A | T |
| 9 | 22489 | C | T |
| 9 | 396818 | T | A |
| 9 | 2024922 | A | T |
| 9 | 2545821 | T | A |
| 9 | 4350798 | T | A |
| 9 | 5907847 | C | G |
| 9 | 6085193 | G | T |
| 9 | 6085745 | G | T |
| 9 | 6170096 | A | C |
| 9 | 6284825 | C | G |
| 9 | 6303440 | C | T |
| 9 | 6531171 | C | T |
| 9 | 7144387 | A | T |
| 9 | 8096883 | A | C |
| 9 | 8281047 | A | G |
| 9 | 8502785 | A | T |
| 9 | 8819684 | G | C |
| 9 | 8951215 | A | G |
| 9 | 10312214 | T | A |
| 9 | 10976584 | T | A |
| 9 | 11034827 | A | T |
| 9 | 12728967 | A | T |
| 9 | 12908317 | C | A |
| 9 | 22737961 | A | T |
| 9 | 22962631 | A | C |
| 9 | 23421278 | G | T |
| 9 | 24442089 | C | T |
| 9 | 24946812 | C | G |
| 9 | 25394584 | C | A |
| 9 | 27373465 | T | A |
| 9 | 27632188 | T | C |
| 9 | 27705808 | G | C |
| 9 | 30971262 | A | T |
| 9 | 34649287 | G | T |
| 9 | 36746523 | G | C |
| 9 | 38283290 | T | A |
| 9 | 38303755 | T | A |
| 9 | 43822900 | G | T |
| 9 | 46169893 | C | A |
| 9 | 48783310 | T | C |
| 9 | 48837610 | G | C |
| 9 | 49167207 | T | C |
| 9 | 50510188 | A | T |
| 9 | 50608174 | T | A |
| 9 | 51889248 | A | T |
| 9 | 53446361 | C | T |
| 9 | 54473422 | T | C |
| 9 | 57423722 | G | T |
| 9 | 57759935 | T | A |
| 9 | 58167234 | G | T |
| 9 | 58196316 | C | T |
| 9 | 58873921 | T | A |
| 9 | 61234598 | G | A |
| 9 | 61633027 | C | G |
| 9 | 62419819 | C | T |
| 9 | 64154886 | G | C |
| 9 | 65022230 | G | T |
| 9 | 65568220 | C | A |
| 9 | 66102261 | C | T |
| 9 | 67364537 | T | C |
| 9 | 67453820 | A | C |
| 9 | 67802827 | G | T |
| 9 | 72185235 | A | G |
| 9 | 72214989 | C | G |
| 9 | 72661128 | A | T |
| 9 | 73128921 | G | T |
| 9 | 73615315 | C | T |
| 9 | 74227474 | G | T |
| 9 | 74271152 | T | G |
| 9 | 76689616 | C | G |
| 9 | 78081584 | T | C |
| 9 | 79915525 | T | A |
| 9 | 80226776 | C | T |
| 9 | 80403874 | G | T |
| 9 | 80822728 | A | T |
| 9 | 82358802 | G | T |
| 9 | 82755959 | T | C |
| 9 | 83825901 | T | A |
| 9 | 83914557 | G | T |
| 9 | 83967698 | G | T |
| 9 | 84489870 | T | A |
| 9 | 84818946 | G | T |
| 9 | 85751054 | A | T |
| 9 | 87574976 | T | A |
| 9 | 90193318 | T | C |
| 9 | 91199084 | T | G |
| 9 | 91545497 | A | T |
| 9 | 91672312 | C | T |
| 10 | 145577 | T | G |
| 10 | 158886 | T | C |
| 10 | 329835 | C | T |
| 10 | 597139 | A | T |
| 10 | 674408 | G | C |
| 10 | 2155132 | T | A |
| 10 | 2739366 | A | T |
| 10 | 3253278 | A | G |
| 10 | 3603916 | T | C |
| 10 | 3927483 | G | A |
| 10 | 4819283 | T | G |
| 10 | 5853045 | G | T |
| 10 | 6266342 | A | G |
| 10 | 7346234 | T | A |
| 10 | 8651604 | C | A |
| 10 | 8883456 | T | C |
| 10 | 9378565 | A | T |
| 10 | 9725842 | C | A |
| 10 | 10383413 | G | C |
| 10 | 16631441 | T | C |
| 10 | 19289974 | A | T |
| 10 | 20492194 | A | C |
| 10 | 21457582 | A | T |
| 10 | 22864140 | A | C |
| 10 | 25039965 | A | C |
| 10 | 25086766 | A | C |
| 10 | 25531489 | T | C |
| 10 | 25864922 | C | T |
| 10 | 27328254 | C | A |
| 10 | 27763453 | G | T |
| 10 | 27962928 | C | T |
| 10 | 30225915 | C | T |
| 10 | 38402054 | T | A |
| 10 | 38533908 | T | G |
| 10 | 38574732 | G | A |
| 10 | 39437321 | T | A |
| 10 | 41401128 | T | A |
| 10 | 41487910 | G | A |
| 10 | 41543675 | T | A |
| 10 | 41548673 | A | G |
| 10 | 41553300 | T | C |
| 10 | 42424784 | A | C |
| 10 | 42537105 | C | T |
| 10 | 43136201 | T | C |
| 10 | 45778734 | C | A |
| 10 | 46736962 | C | A |
| 10 | 48999076 | T | G |
| 10 | 49122076 | T | A |
| 10 | 49373807 | A | G |
| 10 | 50694432 | C | T |
| 10 | 51733753 | G | T |
| 10 | 51741008 | C | T |
| 10 | 52768048 | A | G |
| 10 | 53354548 | T | C |
| 10 | 54355083 | C | A |
| 10 | 54855830 | C | T |
| 10 | 57915440 | T | G |
| 10 | 58018153 | A | C |
| 10 | 58282026 | A | C |
| 10 | 58381189 | T | C |
| 10 | 58415485 | C | A |
| 10 | 59007435 | A | T |
| 10 | 59313767 | T | A |
| 10 | 59401391 | T | A |
| 10 | 59499220 | T | G |
| 10 | 60710452 | A | T |
| 10 | 60825685 | T | C |
| 10 | 61780277 | A | T |
| 10 | 61872089 | A | C |
| 10 | 62812020 | T | A |
| 10 | 62967467 | C | A |
| 10 | 64512437 | A | T |
| 10 | 64908111 | C | T |
| 10 | 65684491 | A | G |
| 10 | 66548980 | A | C |
| 10 | 72176747 | A | T |
| 10 | 73347557 | A | C |
| 10 | 73518730 | A | G |
| 10 | 73720355 | C | T |
| 10 | 74521198 | C | A |
| 10 | 75660598 | A | C |
| 10 | 75999869 | T | C |
| 10 | 76371692 | A | C |
| 10 | 77931720 | T | C |
| 10 | 79507222 | T | A |
| 10 | 80056706 | T | G |
| 10 | 80155677 | T | C |
| 10 | 81071353 | T | C |
| 10 | 82156183 | T | A |
| 10 | 82259969 | G | C |
| 10 | 85674351 | A | G |
| 11 | 592853 | A | C |
| 11 | 929323 | T | C |
| 11 | 2207147 | C | A |
| 11 | 2717874 | G | C |
| 11 | 2777522 | G | T |
| 11 | 4177214 | T | A |
| 11 | 6744875 | G | T |
| 11 | 9251410 | A | T |
| 11 | 11941286 | G | T |
| 11 | 17467994 | C | T |
| 11 | 25094199 | C | T |
| 11 | 26337008 | T | C |
| 11 | 27060755 | A | T |
| 11 | 29744081 | T | A |
| 11 | 30111585 | T | A |
| 11 | 30762644 | T | C |
| 11 | 31438644 | G | C |
| 11 | 31661742 | G | T |
| 11 | 32350096 | C | G |
| 11 | 35044095 | G | T |
| 11 | 35186575 | T | C |
| 11 | 40789689 | A | T |
| 11 | 42678800 | C | G |
| 11 | 46960731 | T | C |
| 11 | 47102533 | A | T |
| 11 | 52978566 | C | T |
| 11 | 54534075 | C | T |
| 11 | 57982857 | A | G |
| 11 | 58829403 | A | G |
| 11 | 60163554 | C | G |
| 12 | 2680954 | T | A |
| 12 | 2871629 | T | A |
| 12 | 3262677 | C | A |
| 12 | 5162741 | A | T |
| 12 | 5317265 | C | A |
| 12 | 5345792 | G | T |
| 12 | 6401775 | A | C |
| 12 | 6695924 | G | A |
| 12 | 7314076 | A | T |
| 12 | 8841802 | A | T |
| 12 | 8909830 | T | A |
| 12 | 9171419 | T | C |
| 12 | 9422334 | G | T |
| 12 | 10480344 | T | A |
| 12 | 11037833 | A | T |
| 12 | 11738989 | C | T |
| 12 | 13703129 | C | G |
| 12 | 13969277 | A | T |
| 12 | 14087670 | A | G |
| 12 | 15443698 | A | T |
| 12 | 15607610 | C | A |
| 12 | 15651347 | T | A |
| 12 | 16814179 | G | A |
| 12 | 18410588 | A | T |
| 12 | 22619113 | A | T |
| 12 | 23936211 | G | T |
| 12 | 24594349 | T | C |
| 12 | 26589369 | G | T |
| 12 | 29688142 | A | T |
| 12 | 32306315 | C | G |
| 12 | 33777461 | A | C |
| 12 | 35834162 | A | C |
| 12 | 37163050 | C | A |
| 12 | 37363816 | A | T |
| 12 | 38414220 | G | C |
| 12 | 39031338 | C | A |
| 12 | 39920583 | A | T |
| 12 | 43442897 | C | G |
| 12 | 45337623 | T | G |
| 12 | 45714709 | T | C |
| 12 | 52006950 | T | G |
| 12 | 56747000 | G | T |
| 12 | 58567896 | C | A |
| 12 | 60475063 | G | C |
| 12 | 62244885 | T | G |
| 12 | 62839009 | A | T |
| 12 | 64734711 | T | A |
| 12 | 67435276 | C | A |
| 12 | 68699681 | T | A |
| 12 | 70963445 | T | A |
| 12 | 71273301 | T | A |
| 12 | 72772061 | C | T |
| 12 | 74025677 | C | A |
| 12 | 74275647 | C | A |
| 12 | 76146918 | A | T |
| 12 | 76502907 | A | T |
| 12 | 76591631 | C | A |
| 13 | 560740 | A | C |
| 13 | 920276 | G | C |
| 13 | 4443063 | T | A |
| 13 | 4746639 | T | A |
| 13 | 6706609 | A | T |
| 13 | 8444783 | A | C |
| 13 | 10452388 | C | T |
| 13 | 11300719 | C | A |
| 13 | 12014356 | G | T |
| 13 | 13490634 | A | T |
| 13 | 18184066 | A | T |
| 13 | 18690449 | T | G |
| 13 | 18960712 | A | T |
| 13 | 20801954 | C | G |
| 13 | 22934107 | G | C |
| 13 | 23730611 | T | A |
| 13 | 25540834 | A | T |
| 13 | 26574689 | A | T |
| 13 | 26696691 | T | A |
| 13 | 27160328 | T | G |
| 13 | 29305959 | T | G |
| 13 | 31785428 | C | T |
| 13 | 35282276 | T | A |
| 13 | 37097194 | T | C |
| 13 | 39362622 | A | G |
| 13 | 49279997 | T | A |
| 13 | 49294338 | A | T |
| 13 | 49442984 | T | A |
| 13 | 49529012 | A | G |
| 13 | 56311848 | T | G |
| 13 | 56475254 | T | G |
| 13 | 59075474 | A | C |
| 13 | 59995317 | A | T |
| 13 | 60181377 | A | C |
| 13 | 61405384 | A | T |
| 13 | 66476444 | T | C |
| 13 | 68552001 | C | G |
| 13 | 71931959 | G | T |
| 13 | 79444425 | T | C |
| 13 | 81523570 | T | C |
| 14 | 4118899 | C | T |
| 14 | 5119880 | A | T |
| 14 | 5884576 | A | G |
| 14 | 10219855 | C | T |
| 14 | 11048855 | A | C |
| 14 | 14301917 | A | T |
| 14 | 14475277 | T | A |
| 14 | 15627434 | T | A |
| 14 | 19824349 | T | G |
| 14 | 20678445 | T | A |
| 14 | 23628189 | A | T |
| 14 | 24308676 | A | T |
| 14 | 24471662 | T | A |
| 14 | 25540330 | A | T |
| 14 | 26189469 | G | C |
| 14 | 27519964 | A | T |
| 14 | 28179756 | T | A |
| 14 | 29587515 | G | T |
| 14 | 31141746 | T | G |
| 14 | 31597147 | A | G |
| 14 | 37609951 | C | T |
| 14 | 37659041 | T | G |
| 14 | 39857436 | A | T |
| 14 | 41298030 | T | A |
| 14 | 42804425 | A | T |
| 14 | 44270924 | T | G |
| 14 | 47850987 | C | A |
| 14 | 48434361 | C | A |
| 14 | 49644143 | C | T |
| 14 | 54465323 | C | T |
| 14 | 54628686 | T | A |
| 14 | 56570328 | T | C |
| 14 | 56887552 | G | T |
| 14 | 57337934 | A | T |
| 14 | 57440978 | G | T |
| 14 | 60059459 | G | T |
| 14 | 60617631 | C | G |
| 15 | 1287087 | T | A |
| 15 | 2439709 | C | T |
| 15 | 2575505 | C | A |
| 15 | 4180140 | T | C |
| 15 | 4382419 | A | T |
| 15 | 4460632 | T | C |
| 15 | 5580473 | T | A |
| 15 | 6620225 | T | G |
| 15 | 6792126 | C | T |
| 15 | 7336653 | C | G |
| 15 | 10772884 | T | C |
| 15 | 10809470 | T | A |
| 15 | 10847563 | T | G |
| 15 | 11442534 | T | C |
| 15 | 11622639 | C | G |
| 15 | 11794262 | T | C |
| 15 | 12148866 | C | G |
| 15 | 14269166 | C | T |
| 15 | 14525359 | T | A |
| 15 | 15393933 | G | T |
| 15 | 16892248 | A | G |
| 15 | 17728166 | T | C |
| 15 | 18286099 | A | T |
| 15 | 19469829 | A | T |
| 15 | 21039584 | T | A |
| 15 | 22306509 | T | C |
| 15 | 23270060 | G | T |
| 15 | 25300899 | C | T |
| 15 | 25375019 | A | T |
| 15 | 26086286 | A | T |
| 15 | 27037347 | T | G |
| 15 | 31801804 | C | A |
| 15 | 34868066 | T | A |
| 15 | 35560877 | C | A |
| 15 | 37956341 | A | T |
| 15 | 38697690 | A | C |
| 15 | 39219306 | A | T |
| 15 | 40370061 | C | A |
| 15 | 44631262 | G | C |
| 15 | 46491877 | T | A |
| 15 | 48981815 | T | A |
| 15 | 49081410 | C | A |
| 15 | 49939328 | C | G |
| 15 | 51268956 | T | A |
| 15 | 52499116 | T | A |
| 15 | 53458704 | G | T |
| 15 | 55144709 | T | A |
| 15 | 56411700 | A | C |
| 15 | 56900733 | A | T |
| 15 | 59016643 | A | T |
| 15 | 61613181 | A | C |
| 15 | 62598522 | T | C |
| 15 | 64032394 | T | G |
| 15 | 64220783 | G | A |
| 15 | 65746179 | C | T |
| 15 | 65890928 | G | C |
| 15 | 66040879 | A | G |
| 15 | 66656297 | T | C |
| 15 | 66968383 | T | A |
| 15 | 67505807 | G | A |
| 15 | 68035772 | T | A |
| 15 | 68052868 | T | A |
| 15 | 69770015 | G | C |
| 15 | 70902850 | T | C |
| 15 | 71488002 | T | A |
| 15 | 74210536 | A | T |
| 15 | 74356960 | A | T |
| 15 | 79324164 | G | A |
| 15 | 80083518 | T | G |
| 16 | 79043 | A | T |
| 16 | 7358560 | A | T |
| 16 | 7492317 | G | T |
| 16 | 7956522 | T | G |
| 16 | 10971126 | A | C |
| 16 | 11954394 | G | T |
| 16 | 13017065 | A | G |
| 16 | 15361589 | T | A |
| 16 | 15639077 | T | C |
| 16 | 24319173 | G | C |
| 16 | 29268631 | C | A |
| 16 | 32307294 | A | T |
| 16 | 34391611 | A | T |
| 16 | 34987924 | T | A |
| 16 | 36107100 | T | C |
| 16 | 36325864 | A | C |
| 16 | 36488372 | G | C |
| 16 | 37828428 | G | T |
| 16 | 38761202 | T | C |
| 16 | 41175887 | T | G |
| 16 | 41628554 | G | T |
| 16 | 43466861 | A | T |
| 16 | 43897232 | T | C |
| 16 | 43959735 | A | G |
| 16 | 43997331 | T | A |
| 16 | 44699819 | T | A |
| 16 | 44805402 | C | A |
| 16 | 45772404 | C | T |
| 16 | 48260772 | T | A |
| 16 | 48888586 | C | A |
| 16 | 48987069 | A | T |
| 16 | 49617504 | T | C |
| 16 | 50124947 | T | A |
| 16 | 50522297 | G | T |
| 16 | 53151984 | T | A |
| 16 | 53524725 | A | T |
| 16 | 54519024 | A | T |
| 16 | 56574067 | T | C |
| 16 | 57162782 | T | C |
| 16 | 57214630 | T | C |
| 16 | 59468806 | A | C |
| 16 | 63645910 | A | C |
| 16 | 63947087 | G | T |
| 16 | 68418003 | C | T |
| 17 | 1990273 | G | C |
| 17 | 5173354 | T | C |
| 17 | 5683808 | A | T |
| 17 | 5762263 | A | T |
| 17 | 12819987 | C | A |
| 17 | 13452653 | A | T |
| 17 | 13947761 | T | A |
| 17 | 14293201 | T | A |
| 17 | 14796293 | G | T |
| 17 | 16099320 | A | C |
| 17 | 16983220 | C | T |
| 17 | 17170300 | C | A |
| 17 | 19056670 | A | T |
| 17 | 19352238 | C | T |
| 17 | 19773004 | T | G |
| 17 | 20207790 | A | T |
| 17 | 22046327 | A | T |
| 17 | 22807406 | A | C |
| 17 | 24133993 | T | C |
| 17 | 25603040 | T | C |
| 17 | 26695151 | T | A |
| 17 | 28368630 | C | A |
| 17 | 30321370 | T | C |
| 17 | 31695600 | C | G |
| 17 | 31952902 | T | C |
| 17 | 33094685 | C | G |
| 17 | 33395607 | G | C |
| 17 | 33526935 | C | T |
| 17 | 33563222 | T | A |
| 17 | 36812317 | C | G |
| 17 | 38193981 | T | C |
| 17 | 38726300 | C | A |
| 17 | 40745186 | A | C |
| 17 | 41132850 | T | A |
| 17 | 41375005 | A | T |
| 17 | 41811537 | A | T |
| 17 | 45989117 | T | A |
| 17 | 47167973 | A | T |
| 17 | 48163274 | A | C |
| 17 | 49842572 | A | C |
| 17 | 50673315 | C | A |
| 17 | 51514715 | T | G |
| 17 | 54494367 | T | A |
| 17 | 55352885 | A | C |
| 17 | 57288152 | A | T |
| 17 | 58614694 | C | T |
| 17 | 60888285 | A | C |
| 17 | 65206888 | T | G |
| 17 | 67080184 | T | C |
| 17 | 67104482 | A | T |
| 17 | 70150456 | A | T |
| 18 | 166359 | T | C |
| 18 | 1153544 | A | T |
| 18 | 2370401 | A | T |
| 18 | 3918438 | T | A |
| 18 | 5978136 | A | T |
| 18 | 7059027 | A | T |
| 18 | 7774352 | A | C |
| 18 | 8189001 | T | A |
| 18 | 10680666 | T | G |
| 18 | 11593566 | A | G |
| 18 | 13365106 | T | C |
| 18 | 17066555 | A | C |
| 18 | 21922520 | A | G |
| 18 | 22622143 | A | T |
| 18 | 25623664 | T | A |
| 18 | 25683401 | T | A |
| 18 | 25991397 | C | A |
| 18 | 27111989 | T | C |
| 18 | 27508044 | C | A |
| 18 | 28996625 | C | G |
| 18 | 33504460 | G | A |
| 18 | 33873054 | T | G |
| 18 | 34091727 | A | T |
| 18 | 34857375 | A | C |
| 18 | 35427047 | T | C |
| 18 | 36712947 | G | A |
| 18 | 36755512 | G | T |
| 18 | 37074885 | T | G |
| 18 | 37076769 | A | T |
| 18 | 37493099 | G | A |
| 18 | 38831658 | T | C |
| 18 | 39327066 | T | A |
| 18 | 40594596 | A | T |
| 18 | 41049779 | C | A |
| 18 | 42867936 | C | A |
| 18 | 49153374 | A | T |
| 18 | 49860211 | A | T |
| 18 | 50369864 | G | C |
| 18 | 52412622 | C | G |
| 18 | 54355431 | A | T |
| 18 | 55886316 | C | T |
| 18 | 56422702 | T | C |
| 18 | 57143823 | T | G |
| 18 | 63939827 | C | T |
| 18 | 64222302 | A | C |
| 18 | 65300562 | C | A |
| 18 | 67480317 | G | C |
| 18 | 68077355 | C | G |
| 19 | 224 | C | T |
| 19 | 1528050 | A | T |
| 19 | 3751759 | A | T |
| 19 | 3757336 | T | A |
| 19 | 4174894 | C | A |
| 19 | 4483660 | A | T |
| 19 | 5467995 | T | A |
| 19 | 5684055 | A | T |
| 19 | 6343536 | T | A |
| 19 | 6408310 | A | C |
| 19 | 7030701 | T | A |
| 19 | 7182497 | C | G |
| 19 | 7373397 | C | T |
| 19 | 9087123 | A | T |
| 19 | 9780209 | G | C |
| 19 | 12130604 | T | A |
| 19 | 14240043 | T | A |
| 19 | 14246431 | T | G |
| 19 | 14364809 | G | A |
| 19 | 14900726 | T | G |
| 19 | 15735809 | G | C |
| 19 | 16274661 | A | T |
| 19 | 16450787 | C | T |
| 19 | 18788695 | C | T |
| 19 | 18896338 | T | C |
| 19 | 19740380 | A | T |
| 19 | 20566777 | T | A |
| 19 | 20572921 | C | G |
| 19 | 20580890 | A | C |
| 19 | 20717367 | G | C |
| 19 | 22053732 | T | A |
| 19 | 22611747 | C | A |
| 19 | 22901011 | T | A |
| 19 | 25449412 | T | C |
| 19 | 25845233 | A | C |
| 19 | 28173837 | G | T |
| 19 | 29242171 | A | T |
| 19 | 30316987 | T | C |
| 19 | 30573344 | G | T |
| 19 | 30763826 | G | T |
| 19 | 31070362 | T | C |
| 19 | 32159686 | T | C |
| 19 | 32264750 | T | G |
| 19 | 33505770 | G | C |
| 19 | 37259445 | A | T |
| 19 | 37718163 | T | A |
| 19 | 40799698 | C | T |
| 19 | 44547029 | A | T |
| 19 | 47359325 | T | C |
| 19 | 54157787 | G | T |
| 19 | 55576481 | C | T |
| 20 | 246600 | A | T |
| 20 | 256906 | A | T |
| 20 | 1919378 | T | A |
| 20 | 2925645 | C | G |
| 20 | 3363081 | G | T |
| 20 | 3378019 | G | T |
| 20 | 4189688 | T | C |
| 20 | 4969390 | C | G |
| 20 | 5195958 | T | C |
| 20 | 5308779 | G | C |
| 20 | 7084658 | C | A |
| 20 | 8866024 | A | G |
| 20 | 10462427 | C | G |
| 20 | 12361102 | T | A |
| 20 | 12505125 | A | C |
| 20 | 17333254 | T | A |
| 20 | 17933281 | T | G |
| 20 | 19910216 | A | C |
| 20 | 21354556 | T | A |
| 20 | 22294585 | A | C |
| 20 | 23435383 | G | T |
| 20 | 25163894 | T | A |
| 20 | 25974585 | G | A |
| 20 | 28530106 | T | A |
| 20 | 28989882 | T | C |
| 20 | 30512835 | A | T |
| 20 | 30715467 | C | T |
| 20 | 33552279 | C | T |
| 20 | 34993282 | C | T |
| 20 | 35755230 | C | T |
| 20 | 37915516 | A | T |
| 20 | 38357281 | T | C |
| 20 | 40810314 | C | T |
| 20 | 41250666 | C | T |
| 20 | 44884788 | A | C |
| 20 | 44936442 | T | C |
| 21 | 2837577 | T | A |
| 21 | 3911258 | G | C |
| 21 | 4978296 | A | C |
| 21 | 7785363 | T | A |
| 21 | 8109789 | G | A |
| 21 | 8373288 | A | T |
| 21 | 8747597 | A | T |
| 21 | 9408551 | C | G |
| 21 | 9704936 | C | A |
| 21 | 10427278 | A | G |
| 21 | 10854820 | A | T |
| 21 | 11093881 | T | C |
| 21 | 12039637 | C | T |
| 21 | 12978238 | G | T |
| 21 | 14654015 | C | T |
| 21 | 17920275 | C | G |
| 21 | 18540772 | A | C |
| 21 | 18910711 | C | A |
| 21 | 23559429 | C | T |
| 21 | 26054948 | T | G |
| 21 | 26653503 | G | C |
| 21 | 27473168 | A | C |
| 21 | 30716617 | C | T |
| 21 | 30906474 | G | A |
| 21 | 32935568 | T | A |
| 21 | 34455203 | C | A |
| 21 | 36047186 | T | A |
| 21 | 36458785 | G | T |
| 21 | 42320768 | T | C |
| 21 | 46996842 | C | A |
| 22 | 611613 | T | C |
| 22 | 1213769 | C | T |
| 22 | 1352933 | A | T |
| 22 | 1450994 | A | T |
| 22 | 3018065 | T | A |
| 22 | 4456195 | A | T |
| 22 | 5645389 | C | T |
| 22 | 5698589 | C | A |
| 22 | 5713929 | T | A |
| 22 | 6264326 | T | C |
| 22 | 6280286 | T | A |
| 22 | 8758294 | A | T |
| 22 | 9263588 | T | C |
| 22 | 9890989 | A | C |
| 22 | 10393408 | A | C |
| 22 | 10919254 | T | A |
| 22 | 12013850 | C | G |
| 22 | 13833245 | A | T |
| 22 | 15777918 | G | T |
| 22 | 21723189 | T | C |
| 22 | 23492210 | G | C |
| 22 | 24986049 | G | T |
| 22 | 26346980 | T | A |
| 22 | 27918305 | A | T |
| 22 | 28465424 | T | A |
| 22 | 29805271 | C | A |
| 22 | 32923865 | A | C |
| 22 | 36233597 | T | C |
| 22 | 37260058 | A | T |
| 22 | 39079729 | T | G |
| 22 | 40477032 | T | A |
| 22 | 42163081 | A | T |
| 22 | 44324545 | G | C |
| 22 | 45078720 | C | G |
| 22 | 46703207 | G | T |
| 22 | 50783542 | T | A |
| 23 | 5735859 | G | T |
| 23 | 6805418 | A | T |
| 23 | 7696707 | T | A |
| 23 | 8342880 | A | T |
| 23 | 8368745 | C | G |
| 23 | 8980832 | G | A |
| 23 | 9735863 | T | G |
| 23 | 10457998 | T | A |
| 23 | 10573611 | A | T |
| 23 | 11913459 | C | T |
| 23 | 12666164 | A | C |
| 23 | 12691877 | T | A |
| 23 | 12780689 | G | A |
| 23 | 13198961 | G | T |
| 23 | 13235433 | T | A |
| 23 | 14876736 | A | T |
| 23 | 15647069 | T | A |
| 23 | 16938726 | C | T |
| 23 | 17128953 | A | T |
| 23 | 17245753 | A | C |
| 23 | 17257076 | T | A |
| 23 | 17861366 | G | C |
| 23 | 17894822 | C | A |
| 23 | 18641630 | A | T |
| 23 | 20562116 | G | T |
| 23 | 20817002 | A | C |
| 23 | 21637601 | A | T |
| 23 | 23434180 | T | G |
| 23 | 24095053 | C | T |
| 23 | 26448883 | T | A |
| 23 | 26966463 | A | T |
| 23 | 27333108 | T | G |
| 23 | 28004052 | T | A |
| 23 | 28246225 | A | C |
| 23 | 31373000 | G | T |
| 23 | 33387188 | A | T |
| 23 | 36756956 | T | A |
| 23 | 38207234 | T | A |
| 23 | 39366338 | G | T |
| 23 | 41378416 | T | A |
| 23 | 42746575 | T | G |
| 23 | 45949255 | A | T |
| 23 | 46167910 | A | C |
| 23 | 47810464 | A | T |
| 23 | 48778379 | A | T |
| 23 | 51468735 | C | T |
| 23 | 53857634 | G | C |
| 23 | 54047402 | T | G |
| 23 | 55137132 | C | A |
| 23 | 55639131 | C | A |
| 23 | 56089058 | T | C |
| 23 | 56394239 | T | G |
| 23 | 60156538 | A | C |
| 23 | 61447803 | G | C |
| 24 | 638364 | T | A |
| 24 | 2734173 | A | C |
| 24 | 3882312 | T | C |
| 24 | 5635787 | T | A |
| 24 | 7365379 | C | T |
| 24 | 12346021 | A | T |
| 24 | 16313834 | G | T |
| 24 | 17328465 | T | A |
| 24 | 17659351 | G | T |
| 24 | 18621657 | C | G |
| 24 | 21759925 | G | T |
| 24 | 21773474 | T | G |
| 24 | 22650153 | T | C |
| 24 | 24306026 | A | C |
| 24 | 24569371 | T | A |
| 24 | 27321940 | G | T |
| 24 | 28031921 | A | T |
| 24 | 30149692 | T | C |
| 24 | 32344418 | T | G |
| 24 | 33439598 | A | C |
| 24 | 38920201 | A | G |
| 25 | 1236269 | C | T |
| 25 | 2482981 | G | T |
| 25 | 2526037 | C | G |
| 25 | 3049279 | C | A |
| 25 | 3488105 | A | T |
| 25 | 6599910 | C | A |
| 25 | 8208957 | A | T |
| 25 | 10864619 | T | A |
| 25 | 10941080 | T | A |
| 25 | 14549015 | T | C |
| 25 | 18912128 | A | C |
| 25 | 20756865 | T | A |
| 25 | 21042045 | A | T |
| 25 | 22051661 | C | T |
| 25 | 22583912 | A | C |
| 25 | 23133553 | T | A |
| 25 | 24082189 | C | G |
| 25 | 24629007 | A | T |
| 25 | 27174214 | C | A |
| 25 | 27303747 | T | G |
| 25 | 30670101 | T | C |
| 25 | 32550817 | G | C |
| 25 | 32699413 | A | T |
| 25 | 35993720 | A | T |
| 25 | 37693869 | A | T |
| 25 | 37796623 | C | T |
| 25 | 38003224 | A | C |
| 25 | 39047419 | C | A |
| 25 | 42291223 | C | G |
| 25 | 44798818 | A | C |
| 26 | 1611853 | G | T |
| 26 | 2312250 | T | A |
| 26 | 2984495 | T | A |
| 26 | 3385965 | A | C |
| 26 | 3660003 | C | A |
| 26 | 3981656 | T | G |
| 26 | 4118220 | C | A |
| 26 | 5027651 | T | C |
| 26 | 5280563 | A | C |
| 26 | 5299108 | T | A |
| 26 | 5764716 | C | A |
| 26 | 6751724 | T | C |
| 26 | 7554858 | T | A |
| 26 | 8366416 | T | G |
| 26 | 8883296 | T | C |
| 26 | 9174283 | A | G |
| 26 | 9821069 | C | A |
| 26 | 10165676 | T | G |
| 26 | 11130499 | A | T |
| 26 | 11947235 | C | A |
| 26 | 12910457 | T | G |
| 26 | 13055878 | T | C |
| 26 | 14441098 | T | C |
| 26 | 15666027 | T | A |
| 26 | 16145664 | C | A |
| 26 | 17663667 | A | G |
| 26 | 19527273 | C | G |
| 26 | 20022737 | C | G |
| 26 | 21354921 | T | C |
| 26 | 22380425 | T | G |
| 26 | 22389758 | A | T |
| 26 | 23298957 | T | A |
| 26 | 23941214 | T | A |
| 26 | 24814472 | C | T |
| 26 | 25130847 | T | C |
| 26 | 27294771 | A | T |
| 26 | 27927279 | C | A |
| 26 | 30531913 | C | T |
| 26 | 31048640 | A | T |
| 26 | 40049784 | A | T |
| 26 | 40190408 | C | A |
| 26 | 41983802 | A | T |
| 26 | 42558828 | A | T |
| 26 | 43273576 | T | C |
| X | 2460503 | G | T |
| X | 3483206 | C | T |
| X | 3506468 | G | T |
| X | 4712449 | C | T |
| X | 5845956 | T | A |
| X | 7887863 | C | T |
| X | 10730205 | C | A |
| X | 19679199 | C | A |
| X | 28808429 | A | C |
| X | 53645426 | C | T |
| X | 61845342 | A | G |
| X | 66905422 | C | A |
| X | 66957662 | C | T |
| X | 71907462 | T | C |
| X | 73227910 | A | C |
| X | 73927156 | G | A |
| X | 88957129 | C | T |
| X | 102851765 | T | C |
| X | 119812482 | C | G |
| X | 132731216 | A | T |

**Supplementary Table 2. Breed associated SNPs identified in the St. Croix breed from the whole genome sequence data.**

| Chromosome | Position | Unique allele | Alternate allele |
| --- | --- | --- | --- |
| 1 | 221897604 | C | A |
| 6 | 52306811 | G | A |
| 8 | 40884967 | G | A |
| 9 | 18117566 | C | A |
| 10 | 49830779 | G | A |
| 10 | 57709685 | G | A |
| 11 | 40538903 | C | T |
| 12 | 56493814 | C | A |
| 13 | 27208267 | G | T |
| 17 | 25116471 | T | A |
| 22 | 28710791 | A | G |
| 1 | 216330513 | T^a^ | C |
| 5 | 48550351 | T^a^ | C |
| 7 | 47802396 | G^a^ | C |
| 7 | 92930155 | G^a^ | T |
| 15 | 9978829 | G^a^ | A |
| 15 | 24294575 | A^~~a~~^ | T |
| 25 | 10864623 | T^a^ | A |
| a. The breed associated SNP was identified in the St. Croix breed when the Katahdin breed was removed from the analysis | | | |
